# Progressive Proximal-to-Distal Reduction in Expression of the Tight Junction Complex in Colonic Epithelium of Virally-Suppressed HIV+ Individuals

**DOI:** 10.1371/journal.ppat.1004198

**Published:** 2014-06-26

**Authors:** Charlotte Y. Chung, Stephanie L. Alden, Nicholas T. Funderburg, Pingfu Fu, Alan D. Levine

**Affiliations:** 1 Departments of Pharmacology, Case Western Reserve University School of Medicine, Cleveland, Ohio, United States of America; 2 Department of Medicine, Case Western Reserve University School of Medicine, Cleveland, Ohio, United States of America; 3 The School of Health and Rehabilitation Sciences, Division of Medical Laboratory Science, The Ohio State University College of Medicine, Columbus, Ohio, United States of America; 4 Departments of Epidemiology and Biostatistics, Case Western Reserve University School of Medicine, Cleveland, Ohio, United States of America; 5 Departments of Pathology, Molecular Biology and Microbiology, Pediatrics, and the Case Comprehensive Cancer Center, Case Western Reserve University School of Medicine, Cleveland, Ohio, United States of America; Vaccine Research Center, United States of America

## Abstract

Effective antiretroviral therapy (ART) dramatically reduces AIDS-related complications, yet the life expectancy of long-term ART-treated HIV-infected patients remains shortened compared to that of uninfected controls, due to increased risk of non-AIDS related morbidities. Many propose that these complications result from translocated microbial products from the gut that stimulate systemic inflammation – a consequence of increased intestinal paracellular permeability that persists in this population. Concurrent intestinal immunodeficiency and structural barrier deterioration are postulated to drive microbial translocation, and direct evidence of intestinal epithelial breakdown has been reported in untreated pathogenic SIV infection of rhesus macaques. To assess and characterize the extent of epithelial cell damage in virally-suppressed HIV-infected patients, we analyzed intestinal biopsy tissues for changes in the epithelium at the cellular and molecular level. The intestinal epithelium in the HIV gut is grossly intact, exhibiting no decreases in the relative abundance and packing of intestinal epithelial cells. We found no evidence for structural and subcellular localization changes in intestinal epithelial tight junctions (TJ), but observed significant decreases in the colonic, but not terminal ileal, transcript levels of TJ components in the HIV+ cohort. This result is confirmed by a reduction in TJ proteins in the descending colon of HIV+ patients. In the HIV+ cohort, colonic TJ transcript levels progressively decreased along the proximal-to-distal axis. In contrast, expression levels of the same TJ transcripts stayed unchanged, or progressively increased, from the proximal-to-distal gut in the healthy controls. Non-TJ intestinal epithelial cell-specific mRNAs reveal differing patterns of HIV-associated transcriptional alteration, arguing for an overall change in intestinal epithelial transcriptional regulation in the HIV colon. These findings suggest that persistent intestinal epithelial dysregulation involving a reduction in TJ expression is a mechanism driving increases in colonic permeability and microbial translocation in the ART-treated HIV-infected patient, and a possible immunopathogenic factor for non-AIDS related complications.

## Author Summary

While antiretroviral therapy for HIV-infected patients is remarkably effective in suppressing viral replication and preventing progression to AIDS, treated patients still have a shorter life expectancy due to increased risks for non-AIDS associated morbidities. Recent data showed that these complications are associated with chronic systemic inflammation, and it is hypothesized that bacterial products breaching the intestinal barrier may cause the inflammation. It is known that HIV induces persistent intestinal mucosal immunodeficiency, but evidence for structural damage to the intestinal epithelium is lacking in the antiretroviral-treated patient population. Here, we characterized the intestinal epithelial damage that leads to increased intestinal permeability in this population. We found that while the colonic epithelial layer is intact microscopically, intercellular tight junctions (TJ) are down-regulated at the transcriptional and translational levels. We observed further that TJ transcripts progressively decrease along the proximal-to-distal HIV gut. Concurrent alterations in the levels of non-TJ epithelial transcripts suggest that epithelial cells in the HIV gut are transcriptionally dysregulated. Our data provide evidence that TJ disruption is a novel mechanism for increasing colonic permeability in the antiretroviral-treated HIV patient, which may then result in systemic inflammation and associated complications.

## Introduction

Chronic systemic inflammation, characterized by increased frequencies of activated B and T cells [1], elevated levels of circulating proinflammatory cytokines and chemokines [2], and more rapid immune cell turnover [3], is a hallmark of HIV/SIV infection and a better predictor of disease progression than plasma viral load [4], [5]. Accumulating evidence suggests that this systemic inflammation plays a role in non-AIDS related comorbidities including cardiovascular diseases [1], [6]–[8], liver diseases [2], [9]–[11], and neurocognitive decline [3], [12], resulting in shortened life expectancy and premature aging in patients treated with long term antiretroviral therapy (ART) [4], [5], [13], [14]. In addition, plasma levels of microbial products, such as lipopolysaccharides (LPS) and bacterial 16s rDNA, are elevated in chronically HIV-infected individuals and associated with markers of immune activation [15]–[17], implicating circulating microbial products, due to microbial translocation, as a major cause of HIV-associated systemic inflammation [18]. An association between circulating microbial products and systemic inflammation has been observed in other disease processes such as inflammatory bowel disease [19], [20] and after laparoscopic surgeries [21], [22]. Moreover, conditioning regimens for stem cell therapy cause gastrointestinal (GI) tract injury that facilitates the translocation of microbial products from the intestinal lumen to systemic circulation, ultimately stimulating the immune system and exacerbating graft-*versus*-host disease [23], [24]. Klatt *et. al.* highlight the association between gut epithelial structural damage, local and systemic microbial translocation, and systemic inflammation, in SIV-naïve pigtail macaques [25], suggesting microbial translocation and systemic inflammation as direct consequences of damage to the GI tract in the absence of chronic viral infection.

The GI tract is a major target site for HIV infection, as the mucosal immune system contains the majority of the body's T cells [26]. In addition, greater than 90% of intestinal CD4+ T cells are CCR5+ [27], providing a large pool of target cells that are preferentially depleted by HIV. Independent of route of transmission, within weeks of HIV or SIV infection, rapid and severe depletion of intestinal lamina propria CD4+ T cells occurs and persists into the chronic phase of the disease [27]–[29], with preferential depletion of the Th17 and Th22 subsets [30], [31]. Significant accumulation of mucosal CD8+ T cells during HIV infection has also been shown [32], [33]; both effects drastically alter mucosal immune homeostasis. Coincident with early mucosal CD4+ T cell loss, gene expression profiling reveals intestinal barrier dysfunction in primary HIV and SIV infection, as exemplified by down-regulation of genes associated with epithelial maintenance and digestive functions [34], [35]. Upregulation of genes with intestinal mucosal protective and regenerative activity in elite controllers [34] confirms the pivotal role intestinal mucosal integrity may play in limiting systemic inflammation and controlling disease progression.

Intestinal barrier dysfunction, long recognized in HIV patients with advanced disease, includes manifestations of pathogen-negative diarrhea and malabsorption [36]. Indirect assessments of intestinal permeability, through measuring urinary excretion of orally consumed oligosaccharides, demonstrate increased small intestinal permeability in symptomatic AIDS patients and some asymptomatic chronic HIV patients, regardless of therapy status [37], [38]. Notably, increased small intestinal permeability did not correlate with intestinal structural change [37], and, through *in vitro* impedance spectroscopy and flux analysis of duodenal biopsies, was suggested to be due to a leak flux mechanism [39], alluding to an intestinal barrier defect as a result of tight junction (TJ) down-regulation. Our recent clinical report demonstrated increased small intestinal and colonic permeability in HIV-infected patients, which was not corrected by ART, further implicating intestinal barrier dysfunction as an ongoing pathophysiological change in ART-treated patients [40]. Our current study, using human intestinal biopsies, extends evidence for intestinal damage in SIV infection of non-human primates [41] and explores the molecular mechanisms behind increased intestinal permeability in ART-treated HIV+ patients. We hypothesize that HIV-associated dysregulation in intestinal epithelial cells will lead to TJ down-regulation, resulting in persistent intestinal barrier dysfunction in the ART-treated patients, contributing to microbial translocation and systemic inflammation.

## Materials and Methods

### Ethics Statement

The University Hospitals Institutional Review Board (IRB) has reviewed the following submission:

Principal Investigator: Dr. Alan D Levine, Ph.D.

Protocol Title: Loss of Intestinal Barrier Function in HIV Infection

UHCMC IRB number: 06-07-31

Submission Type: Continuing Review

Review Type: Full Board

Date of Committee Review: 04/08/2014

As such, the UHCMC IRB has determined that with respect to the rights and welfare of the individuals, the appropriateness of the methods used to obtain informed consent and the risks and potential medical benefits of the investigation, the current submission is acceptable under Federal Human Subject Protection regulations promulgated under 45 CFR 46 and 21 CFR 50 and 56. The current expiration date for this study is 04/07/2015

### Patient Recruitment

Subjects undergoing routine screening colonoscopies were recruited from the Digestive Health Institute and the Special Immunology Unit of the University Hospitals Case Medical Center, Cleveland, OH, with the exclusion criteria of any known or suspected gastrointestinal disease. After written informed consent was obtained, eight pinch biopsies, two each from the terminal ileum, ascending colon, transverse colon, and descending colon, were obtained from thirty-one patients with HIV (median age 51 years, interquartile range [IQR] 50–55 years) and thirty-five healthy controls (median age 56 years, IQR 50–61 years). Peripheral blood was collected immediately following the colonoscopy procedure into EDTA-containing tubes to obtain plasma samples, which were stored at −80°C until assay. Apart from three HIV+ subjects who were simultaneously evaluated for chronic diarrhea, in whom no significant terminal ileal or colonic histopathological findings were identified, there were no reports of diarrhea prior to the colonoscopy preparation regimen for other HIV+ subjects and all controls subjects. HIV+ patients were enrolled notwithstanding their CD4 count and viral load, and all were under ART treatment. Controls were not specifically tested for HIV, but had no reported history of HIV infection. All study protocols were approved by the Institutional Review Board at University Hospitals Case Medical Center.

### Relative Cell Abundance Determined by Nuclear Staining

Formalin-fixed, paraffin-embedded, 5-µm biopsy sections from the ascending, transverse, and descending colon were deparaffinized, rehydrated, mounted in Fluoroshield mounting medium with DAPI (AbCam). Two biopsies per donor from each location were analyzed in a total of five HIV+ patients and five healthy controls. Individual images were obtained on a Leica DMI 6000 B inverted microscope using a 20× objective connected to a Retiga EXI camera (Q-imaging), and composite images of each section were generated through stitching. A threshold intensity for excluding the background was established to specifically analyze nuclear staining. Epithelial cell nuclei and lamina propria cell nuclei were identified manually, after which measurements for the total area they occupy were enumerated using Metamorph Imaging Software (Molecular Devices). The built-in count nuclei application module was used to determine epithelial cell numbers by setting an approximate nuclear width at 3–8 µm. Relative epithelial cell abundance (compared to lamina propria cells) was determined by the ratio of epithelial nuclei area to lamina propria nuclei area. Luminal barrier coverage, defined as the length of the intact epithelial/luminal border relative to lamina propria cell abundance, was designated as the ratio of border length to lamina propria nuclei area. Epithelial cell packing density was calculated as the number of epithelial cells per 100 µm of intact luminal border.

### Confocal Microscopy

Paraformaldehyde-fixed, frozen, 5-µm biopsy sections from the ascending, transverse, and descending colon were blocked with 10% normal goat serum, incubated with rabbit anti-occludin or anti-ZO1 antibody, and detected with chicken or goat Alexa Fluor 488-conjugated anti-rabbit secondary antibody (Invitrogen Life Technologies). Sections were mounted in Fluoroshield mounting medium stained with DAPI (AbCam), and visualized with the Perkin Elmer Ultraview VoX confocal microscope, using an oil-immersion 100× magnification objective lens connected to a Leica DMI 6000 B inverted microscope. *En face* and transverse Z-stack images (0.3 µm thickness) were obtained using Volocity 6.2 (Perkin Elmer). After applying a threshold to eliminate non-specific staining, 3D reconstruction of tight junctions was performed and analyzed using Imaris 3.0 (Bitplane Scientific Software). An average of 7 fields of view on the intestinal surface and 5 fields of view in the crypts were imaged and analyzed for each biopsy obtained, two biopsies per location, from a total of three HIV+ patients and three healthy controls. Average fluorescence intensity for occludin or ZO-1 staining was analyzed for each field of view.

### Real-Time qPCR

Snap-frozen biopsy specimens stored at −80°C were homogenized with a bead beater (Retsch) for 3 min at a frequency of 30 Hz/second to ensure complete homogenization. Total RNA was extracted using the PureLink RNA Mini Kit (Invitrogen Life Technologies, Carlsbad, CA) and quantified with the Nanodrop 2000 (Thermo Fisher Scientific, Wilmington, DE). cDNA was transcribed from 1 µg of total RNA using SuperScript II Reverse Transcriptase (Invitrogen Life Technologies). Transcript levels of human beta-defensin 3 (hBD-3), E-cadherin, and tight junctional proteins occludin, zona occludens 1 (ZO-1), claudin-2, and claudin-4 were determined by SybrGreen-based real-time PCR using CFX96 Real-Time PCR Detection System (Bio-Rad Laboratories). After an evaluation of eight commonly used housekeeping transcripts for genetic stability based on geNormPlus analysis (Biogazelle), β-actin and eukaryotic translation elongation factor 1-alpha 1 (eef1A1) were identified and used as references. Primers used are summarized in **Table 1**. Calibrated normalized relative quantities (CNRQ) of target genes were determined with qBasePlus (Biogazelle) analysis [42].

**Table 1:** Primers used for real time qPCR.

Gene	Primers	
ZO-1	Forward:	AGGGCCCAAGCCTGCAGAGT
	Reverse:	GGAGGGACAGCTGCAGCACC
Occludin	Forward:	CCACGCCGGTTCCTGAAGTGG
	Reverse:	TCACAGGACTCGCCGCCAGT
Claudin-2	Forward:	GGGCACACTGGTTGCCATGCT
	Reverse:	ATGGCCTGGGCAGCCTGGAT
Claudin-4	Forward:	CTCTGGGCGTGCTGCTGTCC
	Reverse:	CGGAGGCCACCAGCGGATTG
hBD-3	Forward:	ATCTTCTGTTTGCTTTGCTCTTCCTGTTTT
	Reverse:	AGCACTTGCCGATCTGTTCCTCCTT
E-cadherin	Forward:	CGACCCAACCCAAGAATCTATC
	Reverse:	AGGTGGTCACTTGGTCTTTATTC
β-actin	Forward:	CAGGCACCAGGGCGTGATGG
	Reverse:	CGATGCCGTGCTCGATGGGG
eef1A1	Forward:	CTTTGGGTCGCTTTGCTGTT
	Reverse:	CCGTTCTTCCACCACTGATT

### Immunoblotting

Total protein was extracted in 60 µl of SDS-RIPA buffer (50 mM Tris pH 8.0, 150 mM NaCl, 0.3% SDS, 1% Triton X, 1 mM EDTA, 1∶100 protease inhibitors) from snap-frozen descending colonic biopsies using a bead beater (Retsch) for 3 min at a frequency of 30 Hz/second, followed by constant agitation for 2 h at 4°C. Proteins were separated by 10% polyacrylamide gel electrophoresis (Invitrogen Life Technologies) and electro-transferred onto nitrocellulose membrane (Invitrogen Life Technologies). After blocking with 5% non-fat milk solution, membranes were probed with rabbit antibodies against glyceraldehyde 3-phosphate dehydrogenase (GAPDH), cytokeratin-18, occludin (AbCam), claudin-2, and mouse antibodies against claudin-4 (Invitrogen Life Technologies) and β-actin (AbCam). After incubation with HRP-conjugated goat anti-rabbit (Thermo Fisher Scientific) or anti-mouse (AbCam) secondary antibody, signals were visualized with enhanced chemiluminescence, using West Pico Supersignal (Pierce). Chemiluminescence for all membranes was detected using Hyblot CL Autoradiography Film (Denville Scientific).

### Densitometric Analysis

The amount of protein in each band was quantified by densitometry using ImageJ (National Institutes of Health). Dilution series were electrophoresed to determine optimal loading amounts for each target protein, to guarantee that bands fell within the linear range of detection. Extracts yielding bands that were too underexposed or overexposed were re-electrophoresed after adjusting the loading volume to obtain bands that were accurately quantified. An inter-gel control sample used to normalize intensity variations between gels was electrophoresed on all gels. The densitometric intensity of each target protein occludin, claudin-2, and claudin-4 was normalized to the intensity of cytokeratin-18 in each extract, to determine epithelial-specific TJ protein levels. Analysis was performed on samples from thirteen HIV+ patients and thirteen healthy controls.

### Measurement of Soluble Inflammatory Markers

Plasma was prepared by centrifugation of EDTA-treated whole blood for 10 min at 1610 g and then frozen at −80°C until assay. Soluble CD14 (sCD14) levels were measured using the Quantikine kit (R&D Systems). Samples were thawed on ice and analyzed in batches in duplicate, background was subtracted, and mean values were reported.

### Measurement of LPS

Plasma samples were diluted to 10% with endotoxin-free water and then heated to 85°C for 15 min to denature plasma proteins. Plasma levels of LPS were quantified with a commercially available Limulus Amoebocyte Lysate assay (QCL-1000, Lonza) according to the manufacturer's protocol. Samples were analyzed in triplicate; backgrounds were subtracted, and mean values were reported.

### Statistical Analysis

For evaluation of the histological samples, statistical analysis was performed utilizing a mixed-effects model that took the repeated measurements from the same individual into account, and unstructured covariance structure was used for the inference. Analyses were performed using SAS (Statistical Analysis System, version 9.2). All other analyses were performed using Prism 5.0 (GraphPad Software). Relative cell abundance, transcript levels in the terminal ileum and colon, as well as protein levels in the descending colon are represented using box-and-whisker plots constructed using Tukey's method, where outliers are noted as distinct data points. Statistical analysis for mRNA, protein levels, and plasma sCD14 levels was performed on all data points, including outliers, via Mann Whitney U test. To analyze for TJ transcript levels *versus* gut location (proximal-to-distal), the results were analyzed using the Kruskal-Wallis test, a non-parametric version of the one-way ANOVA, with a post test adjustment for multiple comparisons to evaluate linear trend. Spearman's rank correlation was calculated for immune activation markers *versus* colonic TJ transcript levels. For samples that were analyzed with multiple comparisons, a False Discovery Rate (FDR) analysis, using the Benjamini and Hochberg's approach [43], was implemented with the SAS procedure PROC MULTTEST. P-values adjusted for FDR are reported. All tests were two-sided and p-values less than 0.07 were considered significant.

### Accession Numbers

We provide accession numbers from Entrez Gene and UniProtKB/Swiss-Prot, for all genes and proteins mentioned in the text, as follows: ZO-1 (Gene ID: 7082; UniProtKB AC: Q07157), occludin (Gene ID: 100506658; UniProtKB AC: Q16625), claudin-2 (Gene ID: 9075; UniProtKB AC: P57739), claudin-4 (Gene ID: 1364; UniProtKB AC: O14493), E-Cadherin (Gene ID: 999; UniProtKB AC: P12830), GAPDH (Gene ID: 2597; UniProtKB AC: P04406), cytokeratin-18 (Gene ID: 3875; UniProtKB AC: P05783), hBD-3 (Gene ID: 414325; UniProtKB AC: P81534), eef1A1 (Gene ID: 1915; UniProtKB AC: P68104), β-actin (Gene ID: 60; UniProtKB AC: P60709).

## Results

### Study Population

**Figure 1:**
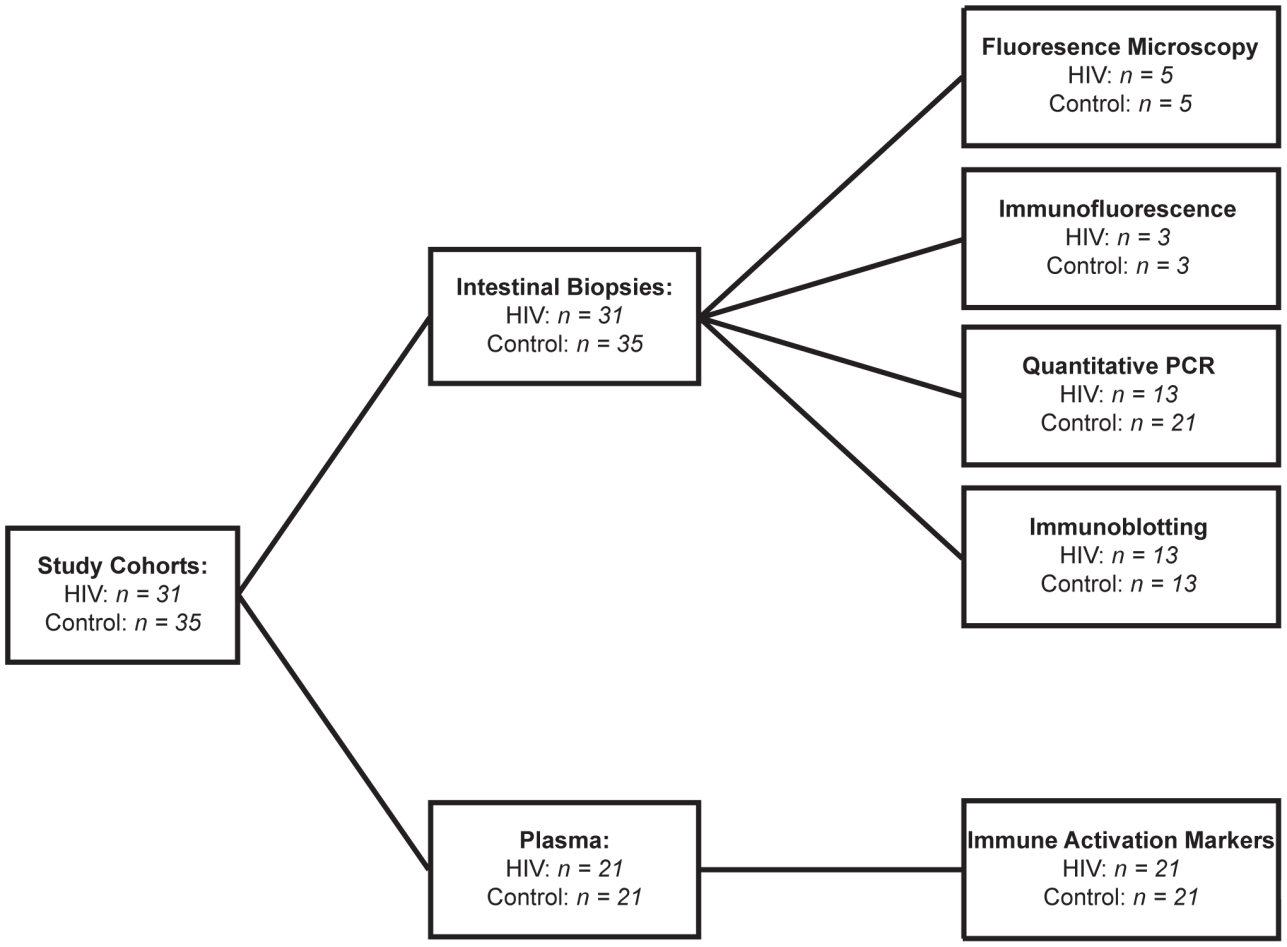
Cohort Diagram illustrating the assignment of intestinal biopsy and plasma samples to each analytical method.

Our clinical study on intestinal barrier integrity in an HIV+ population, in which we measured urinary excretion of orally consumed oligosaccharides [40], revealed increased permeability in the small intestine and colon. These results demonstrated that increased small intestinal permeability is a result of epithelial damage, while colonic paracellular permeability increased without epithelial damage, suggesting a loss in barrier function between intact epithelial cells. Importantly, increases in intestinal permeability were uncorrected in the ART-treated HIV+ patient population. To investigate the mechanism behind the persistent increase in intestinal permeability in ART-treated patients, we obtained intestinal pinch biopsy and plasma samples from thirty-one ART-treated HIV+ patients and thirty-five healthy controls undergoing screening colonoscopies at the Digestive Health Institute at University Hospitals Case Medical Center. Age range was similar between the control and HIV+ cohorts. More males participated than females in both cohorts, with the HIV+ cohort showing a higher percentage of males. HIV+ subjects have been infected for a median of 13.6 years, and reached a median peripheral blood CD4+ T-cell nadir of 176 cells/µl at a median of 6.5 years before the time of biopsy. At the time of biopsy, the median viral load and CD4+ T-cell count of the HIV+ cohort were 48 copies/ml and 569 cells/µl respectively. All but four HIV+ patients had undetectable viral load. All HIV+ patients studied have been under treatment with antiretroviral therapy for a median of 11.2 years, with uninterrupted treatment for a median of 4.1 years prior to study entry. All patients were on a minimum of three antiretroviral drugs, including at least two reverse transcriptase inhibitors and a combination of protease inhibitors and integrase inhibitors, at the time of biopsy. Demographics and clinical parameters of the cohorts are summarized in **Table 2**. Analysis of the plasma levels of immune activation marker sCD14 and LPS, as an indicator of microbial translocation, was performed on twenty-one HIV+ patients and twenty-one healthy controls within our cohorts. Since a limited number of small biopsies were obtained per study subject, we were restricted to undertaking a single molecular or histological analysis on a sample obtained from each subject, with concurrent quantitative PCR and immunoblotting performed on biopsies from three HIV+ and seven control subjects. Subjects in each cohort were randomly assigned to various analyses. **Figure 1** details the breakdown of biopsy and plasma samples from our cohorts into each analytical method.

**Table 2:** Demographics and clinical parameters of study cohorts.

	Control (*n* = 35)	HIV (*n* = 31)
Age, median years (IQR)	56 (50–61)	51 (50–55)
Female sex, %	40	12.9
Ethnicity		
White/Other, %	31.4	54.8
Black, %	60	41.9
Hispanic, %	—	3.2
Unknown, %	8.6	—
Duration of Disease, median years (IQR)	—	13.6 (9.2–17.8)
CD4 nadir, median cells/µl, (IQR)	—	176 (20–281)
Duration since CD4 nadir, median years (IQR)	—	6.5 (3.3–10.6)
Viral load, median copies/ml (IQR)	—	48 (48–50)
CD4 count, median cells/µl (IQR)	—	569 (428–756)
Duration of ART, median years (IQR)	—	11.2 (8.4–13.6)
Duration of continuous ART, median years (IQR)	—	4.1 (2.9–7.6)

### Relative Abundance of Epithelial Cells Is Not Decreased in the Intestine of HIV-Infected Individuals

**Figure 2:**
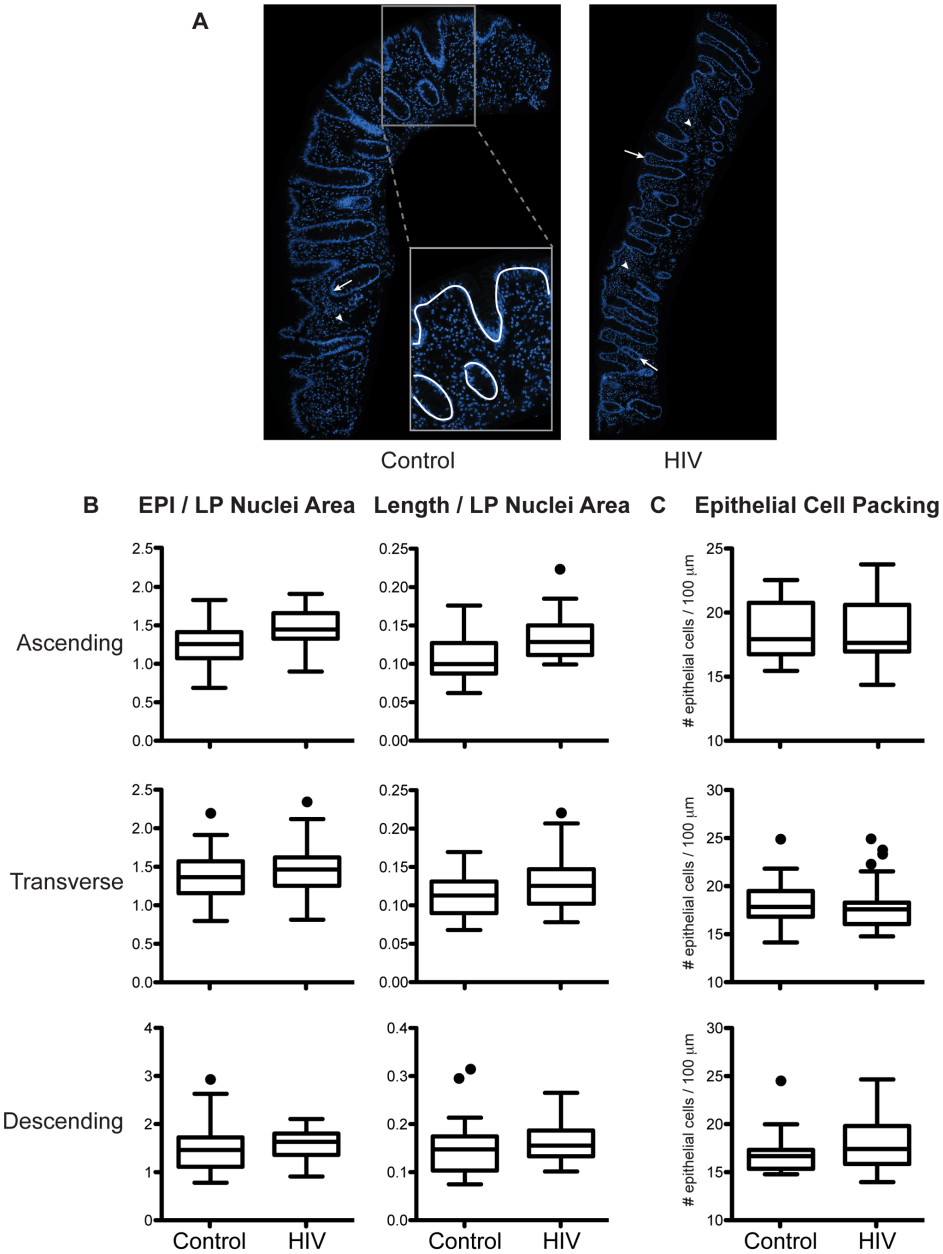
Relative abundance of epithelial cells in intestinal biopsies is not decreased in HIV+ individuals. Fixed paraffin-embedded intestinal biopsy sections were stained with DAPI and imaged. *(A)* Representative images of biopsy sections from a healthy control (left panel) and an HIV+ individual (right panel) are shown with nuclei staining in blue. Abundance of epithelial (EPI) cells, represented by either the area occupied by epithelial cell nuclei (arrow) or the length (Length) of epithelial-luminal border (white line, inset), was compared to the abundance of lamina propria (LP) cells indicated by area occupied by LP cell nuclei (arrowhead). *(B)* Relative abundance of epithelial cells expressed as the ratio between EPI/LP nuclei area or Length/LP nuclei area, and *(C)* epithelial cell packing density denoted by number of epithelial nuclei per 100 µm of epithelial-luminal border, were determined in the ascending, transverse, and descending colon and compared between healthy controls and HIV+ individuals. Box-and-whisker plots were constructed using Tukey's method, where black dots identify the outliers (*n* = 5 for both cohorts).

The intestine is comprised of a one-cell layer thick epithelium, lining the interface with the gut lumen and separating the outside environment from the plethora of immune cells in the lamina propria. To assess an overall loss of epithelial cells as a potential mechanism for increased colonic permeability, we compared the abundance of epithelial cells relative to lamina propria cells in intestinal biopsy samples from HIV+ patients and healthy controls (**Figure 2**). The epithelium in the HIV+ gut is grossly intact and continuous, without areas of focal epithelial cell loss, crypt bifurcation, neutrophil-induced injury, flattened epithelium, or ulceration, either on the luminal surface or in the crypts (**Figure 2A**). Relative abundance of epithelial cells in the HIV+ gut, reflected by the ratio of epithelial cell nuclei to lamina propria cell nuclei, shows no changes in the ascending, transverse, and descending colon (**Figure 2B**). Similarly, considering the extent of intact epithelial-luminal border as a measure of barrier coverage, the relative length of the epithelium in the HIV gut is not altered at all three colonic sites (**Figure 2B**). This evidence for no loss in relative epithelial cell abundance and tissue length in the HIV-infected population is confirmed and extended by finding no change in cell density or packing in the HIV epithelium, as assessed by the number of epithelial cells per 100 µm of epithelium (**Figure 2C**).

These results directly indicate no changes in the abundance of epithelial cells relative to lamina propria cells, not the absolute number of epithelial cells. A previous report demonstrating the restoration of CD4+ T cells and increase in CD8+ T cells, both measured in absolute numbers, in the HIV+ gut mucosa after prolonged ART [32] enables us to conclude that the absolute number of intestinal epithelial cells is not decreased in the colon of HIV-infected individuals. No change in epithelial cell packing in the HIV+ intestinal epithelium also indicates that permeability in the HIV gut is not manifest at the cellular level, suggesting an alternative mechanism, at the molecular level, for the increase in colonic permeability in the ART-treated HIV+ individual.

### No Microscopic Change in the Structure and Subcellular Localization of Intestinal Epithelial Tight Junction Components in HIV-Infected Individuals

Since non-absorbable saccharide probes were utilized in our clinical study, the results reflect an increase in paracellular permeability (extracellular, within the intercellular spaces between epithelial cells), as opposed to solute movement through the transcellular pathway [44]. Paracellular intestinal barrier function is primarily mediated by apically located transmembrane tight junctions (TJs) that seal the intercellular space between adjacent epithelial cells. One potential molecular mechanism for increased colonic intestinal paracellular permeability is the disruption of intercellular TJs. In an intact epithelial cell layer, the space in between individual epithelial cells is sealed by the apical junctional complex composed of the TJ and the subjacent adherens junction, with passage through the TJ being the rate-limiting step of the paracellular transport pathway, and for overall transepithelial solute transport [45]. The TJ is a multi-protein complex composed of transmembrane proteins, including members of the claudin family and TJ-associated marvel proteins such as occludin, which form the intramembranous TJ strands, and intracellular scaffold proteins such as ZO-1, which connect the strand-forming proteins to the cytoskeleton and are important for TJ assembly and regulation [45]. Tissue-specific expression governing the combination of sealing and channel-forming claudin proteins in TJ complexes is the major determinant of distinct barrier properties and selectivity [46], [47].

**Figure 3:**
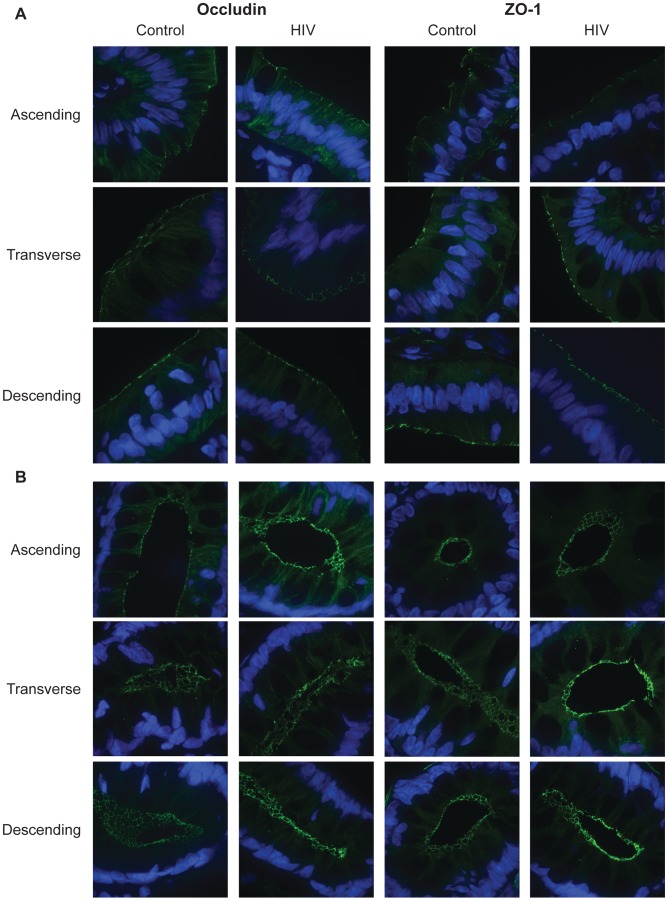
Subcellular localization of tight junctional proteins, occludin and ZO-1, is unaltered in the HIV colon. Fixed, frozen biopsy sections of the ascending, transverse, and descending colon in HIV+ individuals and healthy controls were stained for occludin or ZO-1 (green), and counterstained with DAPI (blue). Representative images at the luminal surface *(A)* and in the crypt *(B)* of the ascending, transverse, and descending colon are shown.

To examine the subcellular localization and organization of the TJ complex in the intestinal epithelium, we studied the distribution of TJ components occludin and ZO-1 in the HIV+ intestine, through high magnification (100×) confocal microscopy. The TJ is located in the apical region of the lateral epithelial cell membrane, close to the luminal surface and away from the basolaterally-situated nucleus and the lamina propria. When the healthy intestinal epithelium is viewed in cross-section, both occludin and ZO-1 appear as distinct dot-like or line-forming structures on the luminal border (**Figure 3A**). When viewed *en face*, TJs, assembled as a ring-like structure in association with the intracellular perijunctional actinomyosin ring, form a ‘chicken wire’ appearance in the crypts of control colonic tissue (**Figure 3B**). Intact TJs are seen in the healthy control population throughout the colon spanning the ascending, transverse, and descending segments. In the HIV+ intestine, no obvious changes are seen in occludin and ZO-1 distribution, both on the intestinal surface and in the colonic crypts (**Figure 3A and 3B**), indicating that epithelial TJ intracellular localization to the lateral plasma membrane is maintained, and that TJs are structurally intact, aligned between epithelial cells, in the ART-treated HIV+ population.

The composition of TJs was also examined in these colonic biopsies. The abundance of occludin and ZO-1 were determined and represented by the average fluorescence intensity in stained ascending, transverse, and descending colonic sections. Average fluorescence intensity is shown in **Tables 3** **and** **4**. No significant difference in occludin intensity in HIV+ samples is seen at the intestinal surface or crypt for all three colonic sites examined. Similarly, no significant difference in ZO-1 abundance is seen on the intestinal surface. In the crypt, significant increases in ZO-1 intensity are observed in the transverse HIV+ colon.

**Table 3:** Average fluorescence intensity for occludin and ZO-1 staining of colonic surface epithelium sections.

Colonic Surface	Occludin				ZO-1			
	Control	HIV	SD#	*p*	Control	HIV	SD#	*p*
Ascending	1085.58	1186.83	152.0	0.676	766.21	710.52	127.0	0.796
Transverse	815.1	674	118.7	0.402	1021.1	1315.2	143.9	0.180
Descending	753.4	1079.6	260.3	0.402	919.7	915.6	76.2	0.958

# SD refers to the standard error of the difference in average fluorescence intensity between the control and HIV cohorts using the mixed-effects model.

**Table 4:** Average fluorescence intensity for occludin and ZO-1 staining of colonic crypt epithelium sections.

Colonic Crypt	Occludin				ZO-1			
	Control	HIV	SD#	*p*	Control	HIV	SD#	*p*
Ascending	1366.18	1616.44	217.4	0.402	756.8	768.7	120.3	0.958
Transverse	849.6	1031.9	162.9	0.402	1177.6	1913.2	132.3	<0.0012
Descending	1102.5	1348.7	166.9	0.402	896.1	1250.4	143.2	0.102

# SD refers to the standard error of the difference in average fluorescence intensity between the control and HIV cohorts using the mixed-effects model.

### Transcripts for Colonic, but Not Terminal Ileal, Tight Junction Proteins Are Down-Regulated in HIV-Infected Individuals

**Figure 4:**
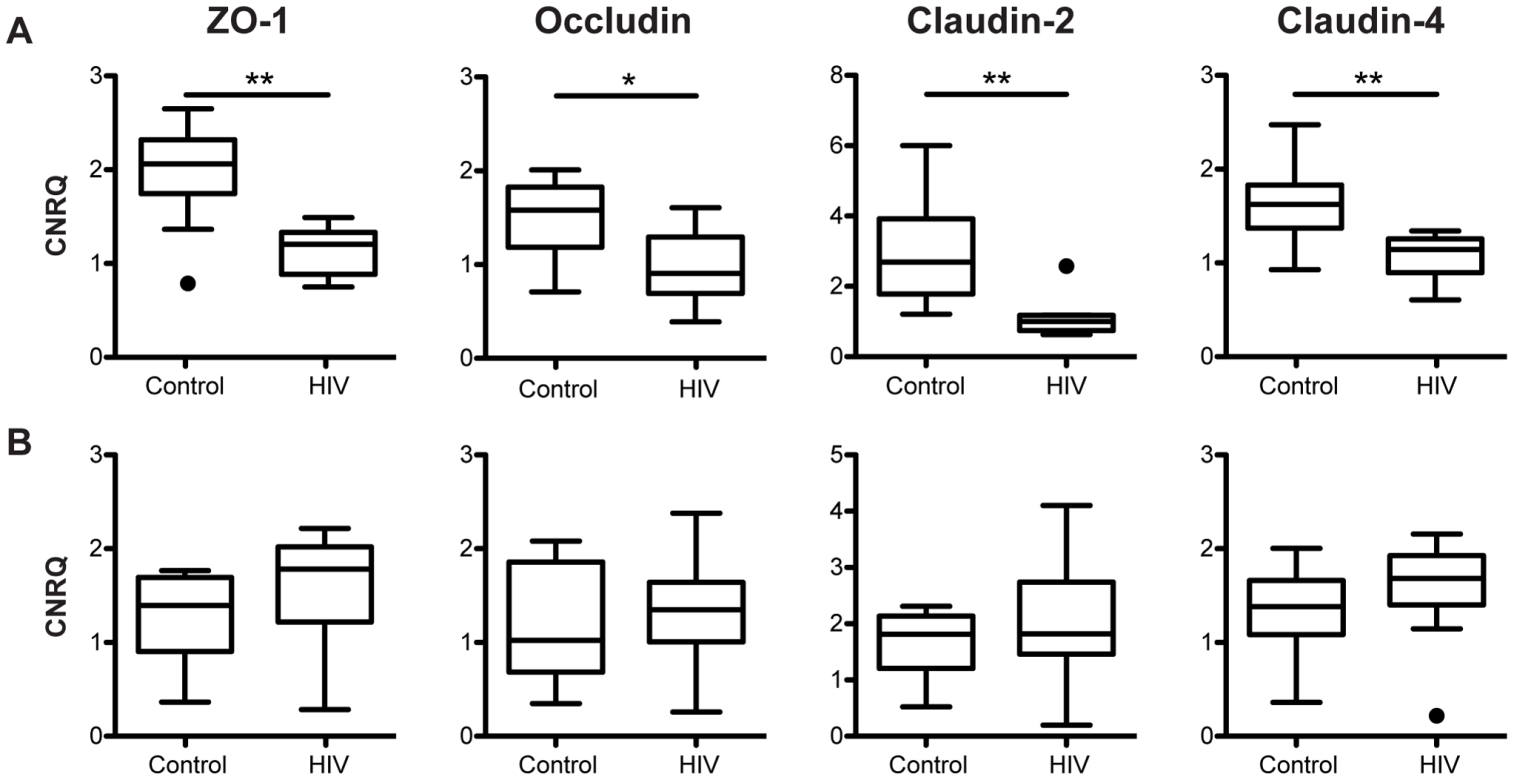
Tight junctional transcripts are decreased in the colon, not the terminal ileum, of HIV+ individuals. Calibrated normalized relative quantities (CNRQ) of ZO-1, occludin, claudin-2, and claudin-4 transcripts were determined in total RNA isolated from *(A)* colonic (*n* = 9 for HIV+, *n* = 13 for healthy controls) and *(B)* terminal ileal (*n* = 10 for HIV+, *n* = 8 for healthy controls) biopsies of HIV+ individuals and healthy controls. Levels are normalized to β-actin and eef1α1 expression. Box-and-whisker plots were constructed using Tukey's method, where black dots identify the outliers. Statistical analysis was performed on all data points, including the outliers (* p<0.05, ** p<0.01 between HIV+ and healthy controls).

To investigate whether the expression of the intestinal epithelial tight junctional complex is regulated in HIV+ patients at the transcriptional level, mRNA concentrations for a panel of TJ proteins were determined in intestinal biopsies through quantitative real-time PCR (**Figure 4**). mRNA levels were quantified using qBasePLUS software, which expresses transcript levels as calibrated normalized relative quantities, calculated based on endogenous levels of β-actin and eef1A1 as controls, selected based on geNorm analysis.

While changes in TJ structure and subcellular localization were undetectable in our system using microscopic immunofluorescence, significant decreases in ZO-1 (p<0.01) and occludin (p<0.05) mRNA expression in the colon (**Figure 4A**) are observed in HIV+ individuals when compared to healthy control subjects. A significant decrease (p<0.01) in the transcript expression level of claudin-2, a cation-selective channel-forming protein, is also observed in the colon of HIV+ subjects, when compared to the expression level in healthy controls. Similarly, a significant decrease (p<0.01) in colonic mRNA expression in the HIV+ patient is detected for another claudin family member, claudin-4 (**Figure 4A**), which functions predominantly as a sealing protein with controversial anion-channel-forming activity. The decrease in TJ mRNA varies from 1.4-fold for claudin-4 to 2.7-fold for claudin-2. We propose that these modest changes in mRNA expression, normalized across a wide stretch of tissue, explain why focal changes in protein expression were undetectable via confocal microscopy examining only a limited number of fields of view. Strikingly, the expression levels of all four TJ mRNAs studied are not changed in the terminal ileum of HIV+ patients as compared to their expression in healthy controls (**Figure 4B**), suggesting an HIV-associated tissue-specific down-regulation of TJ transcripts in the GI tract, seen only in the colon and absent in the terminal ileum. This decrease in TJ mRNA only in the colon is consistent with our clinical study that revealed HIV-associated increased paracellular permeability in the colon and tissue damage in the small intestine [40].

### Expression of Tight Junction mRNA Continuously Decreases along the Proximal-to-Distal Axis in the HIV+ Colon

**Figure 5:**
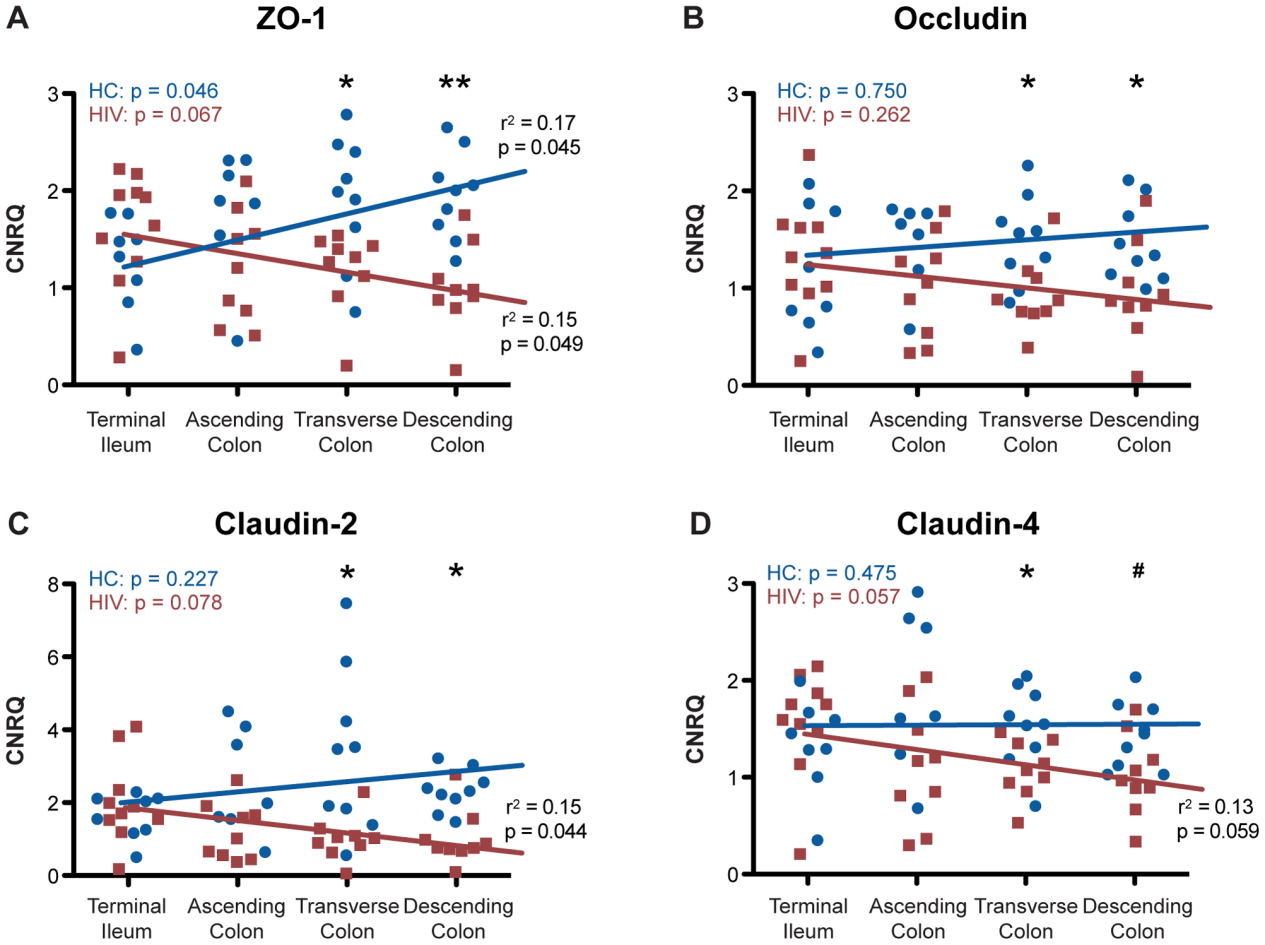
Tight junctional transcript levels in HIV+ individuals decrease progressively from proximal-to-distal colon. Total RNA was isolated from biopsies obtained from the terminal ileum, as well as the ascending, transverse, and descending colon, of HIV+ individuals (red square) and healthy controls (blue circle). CNRQ of *(A)* ZO-1, *(B)* occludin, *(C)* claudin-2, and *(D)* claudin-4 transcripts were compared at each location. The Kruskal-Wallis test, a non-parametric version of the one-way ANOVA, was performed, with p-values for the HIV+ (HIV) and control (HC) cohorts shown in the top left of each panel, after which *r* ^2^ and p-value were determined when appropriate using a post-test for linear trend for each transcript across location in the two cohorts separately. Linear regression is shown as a representation (For HIV+ *n* = 9 at all colonic locations and *n* = 10 at the terminal ileum; for healthy controls *n* = 8 for terminal ileum, *n* = 7 for ascending colon, and *n* = 9 at transverse and descending colon; # p<0.08, * p≤0.05, and ** p<0.01 between HIV+ and healthy controls).

Since small intestinal and colonic biopsies were obtained from four different locations along the GI tract, namely terminal ileum, ascending colon, transverse colon, and descending colon, we are uniquely positioned to investigate whether decreased colonic epithelial TJ mRNA expression in the HIV+ population is differentially distributed relative to anatomical location (**Figure 5**). Using a Kruskal-Wallis analysis with a post test adjustment for linear trend, ZO-1 gene expression shows a significant direct linear increase in transcript level from proximal-to-distal intestine in the healthy control population (p = 0.045). This expression pattern is dramatically reversed in the HIV+ population, where ZO-1 transcript levels demonstrate a significant inverse trend between expression and gut location (p = 0.049) (**Figure 5A**). Comparing ZO-1 transcript levels at each specific intestinal site, we observe a significant decrease in HIV+ individuals as compared to the healthy control population in the more distal portions of the GI tract, namely the transverse (p = 0.05) and descending (p<0.01) colon, consistent with the concept that there is a continual decrease in ZO-1 mRNA expression in the HIV+ population as one travels distally in the colon (**Figure 5A**).

In contrast to ZO-1, occludin, claudin-2, and claudin-4 transcript levels remain relatively constant from proximal-to-distal gut in the healthy control population (**Figure 5B–5D**). While we do not observe a significant linear trend toward reduction in occludin expression toward the distal colon in the HIV+ population, similar to ZO-1 we do find that occludin transcript level is significantly reduced only in the distal, namely the transverse and descending colon in the HIV+ cohort (p<0.05; **Figure 5B**). In congruence with ZO-1 expression in the HIV+ population, claudin-2 and claudin-4 mRNA expression show a significant linear trend toward reduction from the terminal ileum to descending colon (p = 0.044 and p = 0.059; **Figure 5C and 5D**). Examining each colonic location individually, significant decreases in claudin-2 expression are observed in the transverse and descending colon (p<0.05; **Figure 5C**). A similar pattern holds for claudin-4 (**Figure 5D**), with the HIV+ population showing significantly decreased claudin-4 transcript expression in the transverse (p<0.05) and a trend toward a decrease in the descending (p<0.08) colon, while claudin-2 and -4 transcript levels remain unchanged in the terminal ileum and ascending colon (**Figure 5C and 5D**). Overall, HIV infection is associated with a significant modification in the intestinal TJ complex's anatomic expression profile, delineated by a proximal-to-distal decreasing gradient in mRNA expression.

To eliminate the potential effects of differences between the percentages of male *versus* female subjects in the control and HIV+ cohorts, we reanalyzed the expression of intestinal epithelial tight junction transcripts only for the male subjects, which represent the overwhelming majority of HIV+ volunteers. Significant decreases in all four TJ mRNA levels are again observed in the colon of HIV+ males (**Figure S1A**), while transcript levels are not altered in the terminal ileum (**Figure S1B**), replicating the observation for the entire HIV+ and healthy control cohorts. Upon examination of TJ expression levels along the proximal-to-distal axis of the gut for male subjects, we reconfirmed our conclusions on HIV-associated modifications of intestinal TJ complex transcript expression profile, reflected by progressive decreases in transcript levels toward the distal HIV+ intestine. We see an increase in mRNA levels as the location varies from proximal-to-distal for ZO-1 and claudin-2 in healthy males, which is reversed in HIV+ males. For occludin and claudin-4, transcript levels are relatively constant from the terminal ileum to the descending colon of healthy males, while claudin-4 shows a progressive decrease toward the distal colon in HIV+ males. ZO-1, occludin, claudin-2, and claudin-4 median transcript levels are all decreased 1.5 to 2.9-fold in the descending colon of HIV+ males (data not shown).

We also recognize that HIV is a highly heterogeneous disease. While all of our patients are on ART, some of the patients did display a detectable viral load, which may influence intestinal permeability or GI function. With only four patients showing detectable viral load, this study was not powered to compare the fully virally-suppressed group to these four subjects, yet we could eliminate the impact of detectable viral load on TJ transcript expression by removing them from the analysis. Decreases with identical degrees of significance in all four TJ mRNA levels are observed in the colon of virally-suppressed HIV+ subjects (**Figure S2A**), while transcript levels are not altered in the terminal ileum (**Figure S2B**), replicating the observation for the entire HIV+ cohort. Upon examination of TJ expression levels along the proximal-to-distal axis of the colon in fully HIV-suppressed individuals, we found additional decreases in TJ mRNA expression in the ascending colon, which was not seen in the full HIV+ cohort (1.8-fold for ZO-1; 1.7-fold for occludin; 2.3-fold for claudin-2; 1.6-fold for claudin-4).

### Tight Junction Protein Expression Decreases in the Descending Colon of HIV+ Individuals

**Figure 6:**
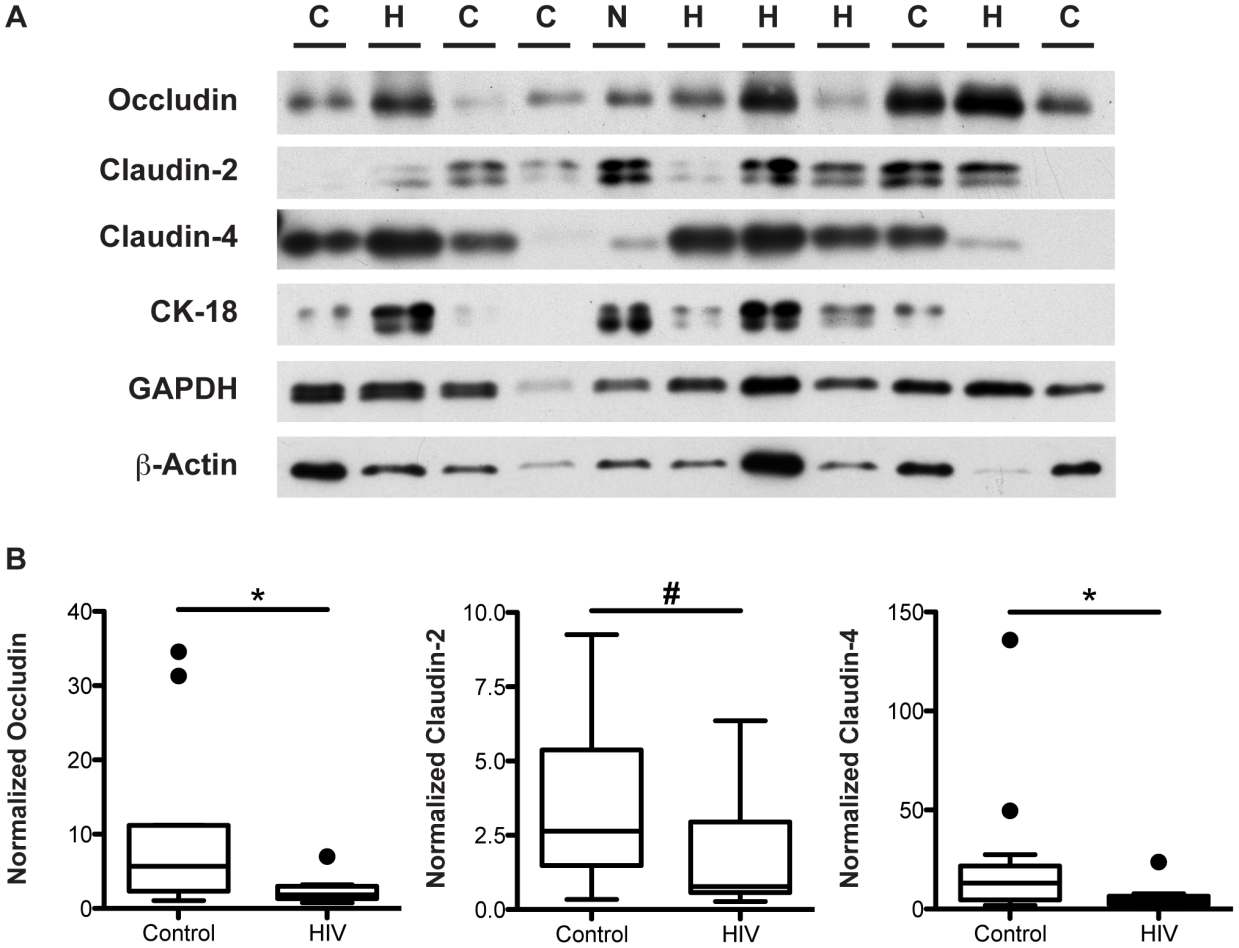
Tight junctional protein levels are decreased in the descending colon of virally-suppressed HIV+ individuals. Total protein lysate extracted from descending colonic biopsies of HIV+ individuals and healthy controls were immunoblotted for occludin, claudin-2, claudin-4, cytokeratin-18, GAPDH, and β-actin. *(A)* A representative blot is shown, with H denoting an HIV+ sample, C denoting a healthy control sample, and N denoting the inter-gel normalizing control sample. *(B)* Specific bands within the linear density range for occludin, claudin-2, and claudin-4 were quantitated by densitometric analysis, and compared between virally-suppressed HIV+ individuals and healthy controls. Target protein levels were normalized against cytokeratin-18 protein levels. Box-and-whisker plots were constructed using Tukey's method, where black dots identify the outliers. Statistical analysis was performed on all data points, including the outliers (For HIV+ *n* = 10 for all proteins; For healthy controls *n* = 13 for occludin and claudin-4, *n* = 12 for claudin-2; # p<0.07, * p<0.05).

To verify the HIV-associated decrease in TJ transcript levels, we measured the protein levels of TJ components occludin, claudin-2, and claudin-4 in the distal colon, the location that showed the greatest reduction in transcript levels in the HIV patient. Total protein lysates from the descending colon of virally-suppressed HIV+ individuals and healthy controls were subjected to immunoblotting (**Figure 6A**) followed by densitometric analysis. Equal loading of samples was verified using GAPDH and β-actin. To accurately quantify the density of each band, samples were electrophoresed twice, varying the loading amounts if needed to obtain a band intensity that fell within the linear range of detection. To examine the TJ protein levels in epithelial cells specifically, levels of each target protein were normalized to the epithelial cell-specific cytokeratin-18 protein, a major cytoplasmic intermediate filament protein expressed in one-layered internal epithelial tissue [48]. Normalized protein levels of occludin, claudin-2, and claudin-4 all show a significant 3.1 to 3.2-fold decrease in the descending colon of the virally-suppressed HIV+ cohort (**Figure 6B**) compared to levels in controls, in agreement with the observed decrease in occludin, claudin-2, and claudin-4 transcript levels in the distal colon of the HIV+ gut. As we described for TJ mRNA, median protein expression levels for occludin, claudin-2, and claudin-4 for the entire HIV+ cohort, as well as in the male HIV+ subjects, are similarly reduced 2.6 to 3-fold in the HIV+ descending colon (**Figures S3 and S4**). Eliminating the limited number of HIV+ patients with diarrhea did not alter these results (data not shown).

### Tight Junction mRNA Down-Regulation in HIV Infection Is a Result of an Overall Change in Intestinal Epithelial Cell Transcriptional Regulation

To investigate whether the transcriptional modification observed in the ART-treated HIV+ population is specific to TJ components, we measured the transcript levels of two epithelial cell-specific proteins that are not part of the TJ complex, namely human beta defensin-3 (hBD-3) and E-cadherin, along the proximal-to-distal gut. hBD-3 is an inducible, broad-spectrum anti-microbial peptide, produced by colonic enterocytes as an innate immune effector to protect against luminal pathogens [49]. E-cadherin is a transmembrane protein component of adherens junctions mediating cell-cell adhesion [50].

**Figure 7:**
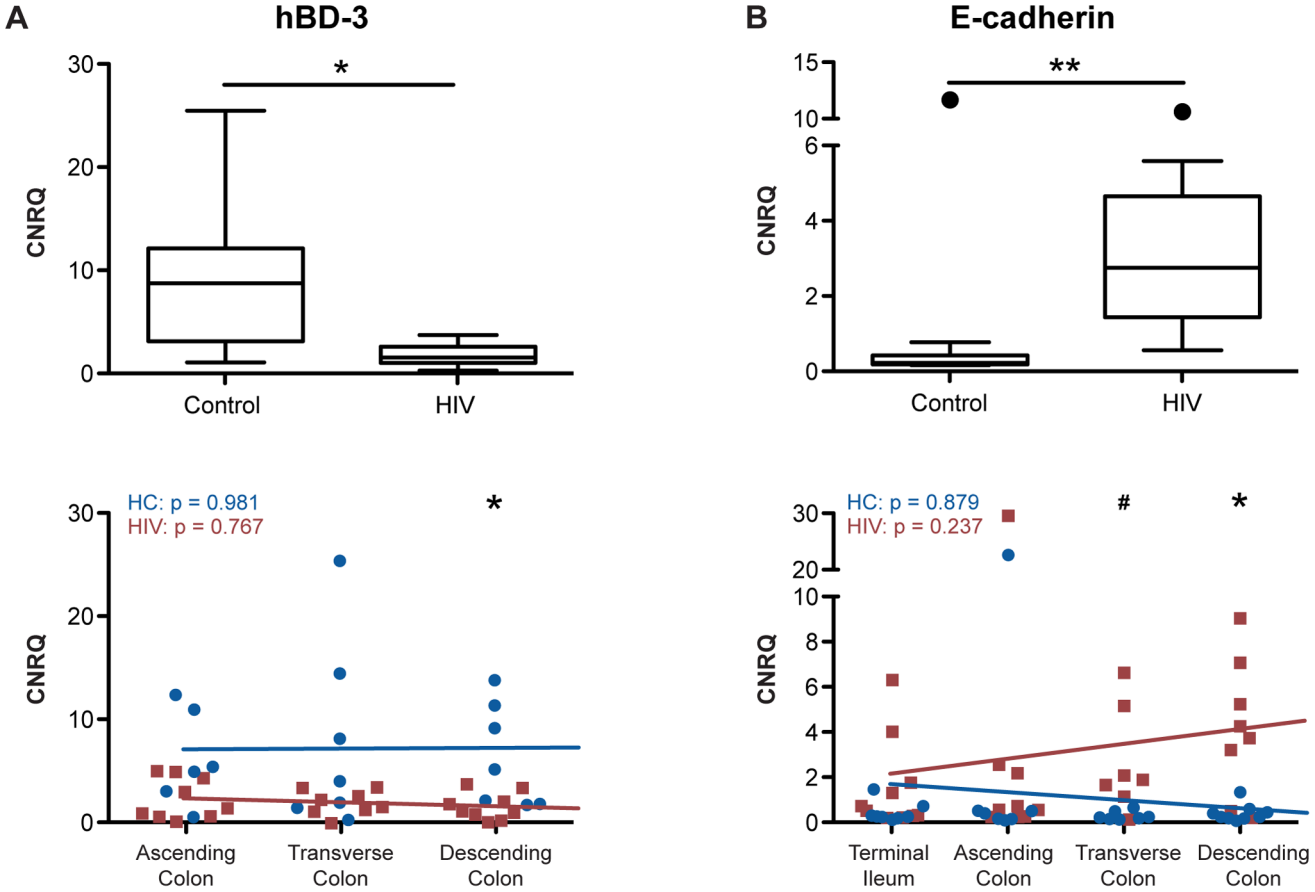
Human β defensin-3 and E-cadherin expression varies differentially from proximal-to-distal HIV+ intestine. Total RNA was isolated from intestinal biopsies, and CNRQ of transcripts for *(A)* human β defensin-3 (*n* = 11 for HIV+, *n* = 12 for control) and *(B)* E-cadherin (*n* = 9 for HIV+, *n* = 13 for control) were measured in the colon of HIV+ individuals and healthy controls (upper panel). Box-and-whisker plots were constructed using Tukey's method, where black dots identify the outliers. Statistical analysis was performed on all data points, including the outliers. Transcript levels were also compared between HIV+ individuals (red square) and healthy controls (blue circle) at each location: terminal ileum, ascending, transverse, and descending colon (lower panel). The Kruskal-Wallis test, a non-parametric version of the one-way ANOVA, was performed for each transcript in the HIV+ (HIV) and control (HC) cohorts separately, with p-values shown in the top left of each panel. Linear regression is shown as a representation (hBD3: For HIV+ *n* = 8 for transverse colon, and *n* = 9 for ascending and descending colon; for healthy controls *n* = 6 for ascending colon, and *n* = 7 for transverse and descending colon; E-cadherin: For HIV+ *n* = 9 for the terminal ileum and ascending colon, *n* = 7 for transverse colon, and *n* = 8 for descending colon; for healthy controls *n* = 9 for the terminal ileum and descending colon, and *n* = 7 for ascending and transverse colon; # p<0.06, * p<0.05, and ** p<0.01 between HIV+ and healthy controls).

Analysis of non-TJ cellular components revealed differing patterns of HIV-associated transcription alteration (**Figure 7**). Similar to that observed for TJ transcripts, transcription of hBD-3 shows a significant down-regulation in the colon of HIV+ individuals (p<0.05), but examination of hBD-3 transcription at specific colonic locations revealed a distinct pattern (**Figure 7A**). Transcription is relatively constant in the healthy control population along the ascending, transverse, and descending colon, while in the HIV+ population, although a significant hBD-3 transcriptional down-regulation is observed only in the distal descending colon (p<0.05), there is no significant linear trend between hBD-3 transcript level and anatomical location (**Figure 7A**). E-cadherin, on the other hand, shows transcriptional up-regulation in the HIV+ colon (p<0.01), compared to levels in the uninfected colon, in stark contrast to other transcripts studied (**Figure 7B**). Comparing between the healthy controls and the HIV+ population at each gut location, we observed a significant upregulation of E-cadherin transcript levels at the transverse (p<0.06) and descending (p<0.05) HIV+ colon. In the terminal ileum and ascending colon, E-cadherin transcript levels are unchanged. E-cadherin transcript levels do not vary with location in the healthy or HIV+ gut. (**Figure 7B**). Identical results for hBD-3 and E-cadherin transcript levels in the colon are observed when only male donors or virally-suppressed populations were examined separately (**Figures S5 and S6**).

### Microbial Translocation and Immune Activation Correlated with a Decrease in Colonic TJ Transcript Expression

**Figure 8:**
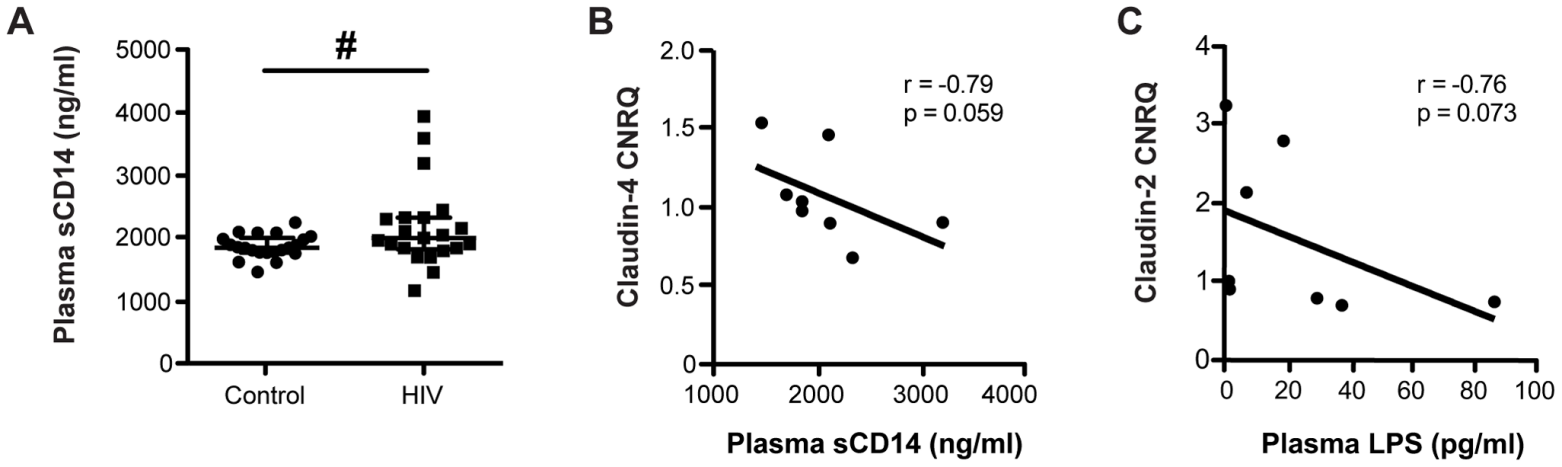
Microbial translocation and systemic immune activation marker levels inversely correlate with colonic TJ transcript levels. *(A)* Plasma samples from HIV+ individuals and healthy controls were thawed and analyzed by ELISA to measure levels of soluble CD14 (sCD14). Medians are denoted, with bars representing the interquartile range (*n* = 21 for both cohorts; # p<0.07). Plasma levels of *(B)* the immune activation marker sCD14 and *(C)* the microbial product LPS inversely correlate with transcript levels of claudin-4 and claudin-2, respectively, in the descending colon of subjects from both the HIV+ and healthy control cohorts. Spearman's rank test was used to determine correlations. The black lines represent the linear regression estimate.

It has been previously reported that circulating levels of the microbial cell wall constituent, LPS, is elevated in both ART-treated and untreated HIV+ patients [15]–[17], and we hypothesize that the accessibility of translocated luminal microbial products is mediated by a decrease in intestinal TJ expression. To confirm that an inflammatory mediator indicative of LPS exposure is elevated in our HIV+ cohort, we measured plasma levels of soluble CD14 (sCD14), an LPS co-receptor that promotes its binding to Toll-like receptor-4 and is shed from activated monocytes. Levels of sCD14 were elevated in samples from HIV+ patients compared to healthy controls (**Figure 8A**). Furthermore, there are inverse correlations that trend toward significance between levels of LPS or sCD14 and claudin transcript expression in the descending colon of both HIV+ and healthy control subjects (r = −0.79, p = 0.059 for claudin-4 vs. sCD14; and r = −0.76, p = 0.073 for claudin-2 vs. LPS; **Figures 8B and 8C**), demonstrating a direct link between TJ gene expression in the distal colon and immune activation in HIV infection.

## Discussion

Microbial translocation from the gut, originating from the enormous quantity of intestinal commensal bacteria, is implicated as a major driver of the chronic systemic inflammation that not only predicts pathogenic HIV disease progression and poor response to ART, but, more importantly, may mediate the immunopathogenesis of non-AIDS morbidities, including cardiovascular, liver, and neurocognitive diseases, that shorten the life expectancy of long-term ART-treated HIV-infected individuals [51], [52]. Elevated microbial translocation is attributed to the simultaneous effects of intestinal mucosal immunodeficiency and disruption to the epithelial barrier, a hypothesis confirmed in pathogenic SIV infection [41]. Structural damage to the intestinal epithelium has been demonstrated in ART-naïve HIV-infected population [53], [54], and we now provide the first direct molecular evidence of gut barrier breakdown in virally-suppressed HIV+ patients, corroborating our clinical data demonstrating persistence of increased intestinal permeability in the ART-treated population [40]. Intestinal epithelial disruption is restricted to the colon and manifests at the molecular level as a down-regulation of the TJ components ZO-1, occludin, claudin-2, and claudin-4, via transcriptional control. The colonic epithelium remains grossly intact, and the packing and relative abundance of epithelial cells are maintained. Moreover, we observed a progressive decline in TJ expression along the proximal-to-distal axis of the HIV+ colon, in contrast to the relatively flat or increasing gradients observed in the healthy intestine. Finally, concurrent alterations in the transcriptional pattern of non-TJ epithelial-specific genes suggest that tight junctional down-regulation in the HIV+ gut occurs as part of an overall intestinal epithelial disruption through modified regulation of transcription.

The dramatic and rapid depletion of CD4+ T cells from the intestinal mucosa during HIV and SIV infection led to the speculation that injury to the immune component of the intestinal lamina propria is permissive for increased translocation of microbial products into systemic circulation [18]. Indeed, mucosal immunodeficiency begins in the early phase of HIV or SIV infection and is characterized by a profound and selective depletion of CD4+ T cells within days of infection [55] and preferential loss of IL-22 and IL-17 producing T cells [31], [56], impaired neutrophil recruitment and macrophage phagocytic function [41], and local mucosal inflammation [52], [57], [58]. Recent literature indicates that mucosal immunodeficiency and structural epithelial deterioration concurrently drive microbial translocation and HIV progression [59]. Intestinal epithelial disruption occurs in early SIV infection through epithelial cell apoptosis, secondary to interactions with the intestinal epithelial cell-associated alternative SIV coreceptor GPR15/Bob [60]. In humans, epithelial dysregulation begins in primary HIV infection and persists into the chronic phase, with down-regulation of genes involved in epithelial maintenance, growth and differentiation, as well as metabolic and digestive functions [34], [61]. Our results, while highlighting the significant decrease in TJ mRNA and protein expression in chronic HIV infection, reveal a broader change in intestinal epithelial cell transcriptional regulation, even in the setting of viremic control. Our findings are in agreement with the notion of overall epithelial dysregulation in chronic HIV infection, rather than a specific disturbance in TJs, leading to structural deterioration of the epithelial barrier on the molecular level.

Evidence for epithelial barrier disruption contributing to SIV pathogenesis has been demonstrated in the non-human primate model. A comprehensive survey of the entire colonic tissue revealed epithelial barrier breakdown in chronic (non-AIDS and AIDS), untreated SIV-infected rhesus macaques, varying from multifocal colonic epithelial disruptions to epithelial loss and overt ulceration [41]. This breakdown in the epithelium is associated with *in situ* LPS infiltration into the lamina propria and local immune activation [41]. Our results demonstrating an intact intestinal epithelium at the endoscopic and light microscopic level with no decrease in epithelial barrier coverage in the HIV+ colon is more in agreement with a recent study showing microbial translocation *in situ* with little morphological evidence of human intestinal epithelial breaches [54]. Together these reports suggest distinct mechanisms between humans and primates for intestinal barrier loss. However, we acknowledge that the studies in humans are limited by a random sampling of the colonic mucosa via a restricted number of biopsy samples. Discrete sites of epithelial barrier loss or ulceration, not visible to the clinician, may have been missed in our study. Thus, we do not dismiss the possible contribution of epithelial barrier breakdown, at the cellular level, to HIV-associated microbial translocation, in addition to epithelial TJ down-regulation.

While global intestinal epithelial cell function is compromised during HIV infection, in this report we focus on decreased TJ expression in the HIV+ intestinal epithelium as promoting the translocation of microbial products to the lamina propria and systemically, extending *in vitro* evidence that demonstrated TJ downregulation as a response of genital and intestinal mucosal epithelium to direct HIV-1 exposure [62]. In contrast, Smith *et al.* [54] reported increased levels of claudin-2 protein in the ileum and rectum during chronic HIV infection. Similarly, Epple *et al.* [53] demonstrated mucosal barrier defects in the duodenum of HIV-infected individuals including increased mannitol permeability, decreased claudin-1, and increased claudin-2 protein expression. These earlier results were obtained from untreated viremic patients. Our findings are complementary, except for claudin-2, and expand the current understanding of HIV-associated TJ disruptions in patients with effective viral suppression. While Epple *et al.* showed that duodenal mucosal barrier changes are reversed in the small intestine of antiretroviral-treated patients, we systematically probed for TJ disruptions along the length of the colon, highlighting the greatest down-regulation of TJs, both at the transcriptional and translational levels, in the most distal, descending portion of the colon. These distinct differences in response of the small and large intestines to HIV infection and ART highlight the contributions of apoptosis in the former and paracellular permeability in the latter [25], [40], [60], suggesting unique pathologies of HIV damage along the alimentary canal, and must always be tempered by the sample size and heterogeneity of the populations studied.

As noted above, our previous clinical study indicates that the increase in small intestinal permeability seen in virally-suppressed, HIV-infected individuals is primarily a result of epithelial cell damage. Consistent with these clinical results, in this report we find that the TJ complex is down-regulated only in the colon, not the small intestine. In the pre-antiretroviral era, advanced HIV disease was associated with small intestinal structural defects, including villous atrophy and crypt hyperplasia [63]. More recently, multiple studies have shown correlation between disease progression, circulating microbial products due to translocation, and plasma levels of intestinal fatty acid binding protein (I-FABP) [64]–[66], which is a marker of small intestinal epithelial cell apoptosis [67]. These results collectively stress the importance of epithelial cell damage through apoptosis as the predominant mechanism for loss of small intestinal barrier integrity.

We also present results pertaining to perturbations in the expression pattern of various epithelial-specific transcripts along the proximal-to-distal axis of the HIV and healthy gut, from the terminal ileum to the descending colon. The distally increasing pattern observed for ZO-1, for instance, in the healthy population is consistent with findings from gene expression mapping along the normal colon identifying transcripts that are differentially expressed from proximal-to-distal segments, a subset of which demonstrates a gradual monotonic change in expression levels, characteristically an increase toward the distal colon [68]. The other subset identified consisted of transcripts with a dichotomous proximal *versus* distal colon expression pattern. We postulate that such gene expression profiles along the longitudinal axis of the gut is programmed by embryonic development and established by interactions with the external environment. The small and large intestines are distinct organs in the alimentary canal, with unique functions of digestion and absorption of nutrients, and reabsorption of water and electrolytes, respectively. Consecutive anatomical regions within the colon also perform distinct functions, with the proximal portion relatively more involved in solidification of fecal contents as compared to the distal portion, which is responsible for the transient storage of feces [69]. Indeed, such functional differences mirror and are determined by the various intrinsic differences between the terminal ileum, proximal, and distal colon in terms of embryologic origin, morphology, and proliferative capacity [70]. Interactions with luminal content, dependent on diet, and microbiota further modify the epithelium, the degree to which is dependent on colonic transit time, shown to be slowest in the proximal colon [71]. In addition, luminal microbiota shifts along the length of the colon in quantity and diversity [72], and metaproteome analysis of the colonic mucosal-luminal interface demonstrate significant anatomic region-related differences in host-microbial interactions [73]. Such variations have pathological consequences, with the classic “two-colon concept” of colorectal carcinoma describing striking differences in clinical, molecular, and epidemiological features of tumors in the proximal and distal colon [74], and more recent data revealing a gradual increase in the frequency of the CpG island methylator phenotype, microsatellite instability, LINE-1 methylation, as well as BRAF, KRAS, and PIK3CA mutations in tumors along the bowel at different colonic subsites [75].

We acknowledge that our observed HIV-associated intestinal TJ downregulation and gene expression pattern alterations are likely a result of complex interactions between the gut environment and intestinal epithelial cells. While we cannot fully address all of the sources of heterogeneity with this limited sample size, we sought to minimize the potential impact of identifiable confounders, while acknowledging that our methodology involves an appreciable number of multiple comparisons, thus reducing our statistical power. While there can be small differences in intestinal transcript expression patterns in females *versus* males [69], our conclusions are maintained in age-matched male subjects in our cohorts, eliminating age and gender as confounding factors for the observed HIV-associated changes. Using a similar strategy, we were able to eliminate detectable viral load as a potential confounder. Gene expression profile along the colonic mucosa is modulated after colectomy surgery to correspond to the new (proximal or distal) location [69], providing evidence that colonic transcript expression levels along the proximal-to-distal axis are responsive to pathophysiological perturbations or insults. In light of the importance of the specific microbiome composition on maintaining the gut's structural barrier, as well as local and systemic immunity [76], alterations of the luminal microbiome (dysbiosis) in association with HIV infection [52], [77], [78] in a colonic subsite-specific manner would influence local epithelial function and transcriptional activity, resulting in differential proximal-to-distal TJ component expression patterns between the HIV+ and healthy colon. It is important to recognize that luminal and mucosa-associated enteric bacteria represent two distinct populations, with the mucosa-associated population displaying local heterogeneity [72], [79]. Studies on stool microbiome reveal increased diversity and altered composition of microbiota in the HIV gut – changes that persist in the ART-treated patient [78], [80]. Similarly, dysbiotic mucosal-adherent microbiota are observed in the ART-treated HIV-infected patient [81], paving the way to a systematic study of dysbiosis along the longitudinal axis of the HIV gut, that may directly influence intestinal epithelial cell metabolism and function. In addition, expansion of the enteric virome, associated with a previously undescribed set of viruses, in pathogenic SIV infection [77] raises the possibility of viral contributions to HIV progression and intestinal pathology.

HIV-associated enteric dysbiosis may also have consequences on the lamina propria T cell populations. Total bacterial load in the stool of HIV-infected subjects negatively correlates with duodenal T cell activation, while the proportions of *Enterobacteriales* and *Bacteriodales* are associated with duodenal CD4+ T cell loss [80]. Enrichment of gut bacteria that catabolize tryptophan via the kynurenine pathway [81] may inhibit the differentiation of Th17 cells, a T cell subset important in maintaining mucosal immunity shown to be depleted in the HIV lamina propria. Recent data from our laboratory demonstrate that T cell activation can modulate intestinal epithelial barrier permeability, suggesting possible contributions of lamina propria T cells on intestinal epithelial TJ regulation.

In the ART era, non-AIDS associated complications are now a major clinical focus and concern. Systemic inflammation, possibly initiated by circulating microbial products from the gut lumen, is a likely contributing factor to HIV morbidity. Our demonstration of inverse correlations between markers of immune activation and TJ transcript levels and a proximal-to-distal gradient of decreased TJ expression in the HIV colon provide a needed mechanism for increased intestinal permeability in the well-controlled, ART-treated HIV-infected patient, which contributes to microbial translocation and systemic inflammation.

## Supporting Information

Figure S1 **Tight junctional transcripts are decreased in the colon, not the terminal ileum, of male HIV+ individuals.** Calibrated normalized relative quantities (CNRQ) of ZO-1, occludin, claudin-2, and claudin-4 transcripts were determined in total RNA isolated from *(A)* colonic (*n* = 8 for HIV+, *n* = 7 for healthy controls) and *(B)* terminal ileal (*n* = 8 for HIV+, *n* = 6 for healthy controls) biopsies of male HIV+ individuals and healthy controls. Levels are normalized to β-actin and eef1α1 expression. Box-and-whisker plots were constructed using Tukey's method, where black dots identify the outliers. Statistical analysis was performed on all data points, including the outliers (* p<0.05, ** p<0.01 between HIV+ and healthy controls).
(TIF)

Figure S2 **Tight junctional transcripts are decreased in the colon, not the terminal ileum, of virally-suppressed HIV+ individuals.** Calibrated normalized relative quantities (CNRQ) of ZO-1, occludin, claudin-2, and claudin-4 transcripts were determined in total RNA isolated from *(A)* colonic (*n* = 8 for HIV+, *n* = 13 for healthy controls) and *(B)* terminal ileal (*n* = 7 for HIV+, *n* = 8 for healthy controls) biopsies of virally-suppressed HIV+ individuals and healthy controls. Levels are normalized to β-actin and eef1α1 expression. Box-and-whisker plots were constructed using Tukey's method, where black dots identify the outliers. Statistical analysis was performed on all data points, including the outliers (* p<0.05, ** p<0.01 between HIV+ and healthy controls).
(TIF)

Figure S3 **Tight junctional protein levels are decreased in the descending colon of HIV+ individuals.** Total protein lysate extracted from descending colonic biopsies of HIV+ individuals and healthy controls were immunoblotted for occludin, claudin-2, claudin-4, cytokeratin-18, GAPDH, and β-actin. Specific bands within the linear density range for occludin, claudin-2, and claudin-4 were quantitated by densitometric analysis, and compared between HIV+ individuals (including those with detectable viral load) and healthy controls. Target protein levels were normalized against cytokeratin-18 protein levels. Box-and-whisker plots were constructed using Tukey's method, where black dots identify the outliers. Statistical analysis was performed on all data points, including the outliers (*n* = 13 for both cohorts, except *n* = 12 for healthy controls in claudin-2; # p≤0.07).
(TIF)

Figure S4 **Tight junctional protein levels are decreased in the descending colon of male HIV+ individuals.** Total protein lysate extracted from descending colonic biopsies of male HIV+ individuals and healthy controls were immunoblotted for occludin, claudin-2, claudin-4, cytokeratin-18, GAPDH, and β-actin. Specific bands within the linear density range for occludin, claudin-2, and claudin-4 were quantitated by densitometric analysis, and compared between male HIV+ individuals and healthy controls. Target protein levels were normalized against cytokeratin-18 protein levels. Box-and-whisker plots were constructed using Tukey's method, where black dots identify the outliers. Statistical analysis was performed on all data points, including the outliers (*n* = 11 for HIV+, *n* = 7 for healthy controls; # p<0.09).
(TIF)

Figure S5 **Human β defensin-3 and E-cadherin expression varies differentially in the colon of HIV+ males.** Total RNA was isolated from intestinal biopsies, and CNRQ of transcripts for *(A)* human β defensin-3 (*n* = 9 for HIV+, *n* = 7 for healthy controls) and *(B)* E-cadherin (*n* = 8 for HIV+, *n* = 7 for healthy controls) were measured in the colon of male HIV+ individuals and healthy controls. Box-and-whisker plots were constructed using Tukey's method, where black dots identify the outliers. Statistical analysis was performed on all data points, including the outliers. (** p<0.01 between HIV+ and healthy controls).
(TIF)

Figure S6 **Human β defensin-3 and E-cadherin expression varies differentially in the colon of virally-suppressed HIV+ individuals.** Total RNA was isolated from intestinal biopsies, and CNRQ of transcripts for *(A)* human β defensin-3 (*n* = 9 for HIV+, *n* = 12 for healthy controls) and *(B)* E-cadherin (*n* = 8 for HIV+, *n* = 13 for healthy controls) were measured in the colon of virally-suppressed HIV+ individuals and healthy controls. Box-and-whisker plots were constructed using Tukey's method, where black dots identify the outliers. Statistical analysis was performed on all data points, including the outliers (* p<0.05 between HIV+ and healthy controls).
(TIF)
