# Supplementary figures and images for: Progressive Proximal-to-Distal Reduction in Expression of the Tight Junction Complex in Colonic Epithelium of Virally-Suppressed HIV+ Individuals
Source: PLoS Pathog. 2014 Jun 26;10(6):e1004198. doi: 10.1371/journal.ppat.1004198 (PMC4072797; doi:10.1371/journal.ppat.1004198)

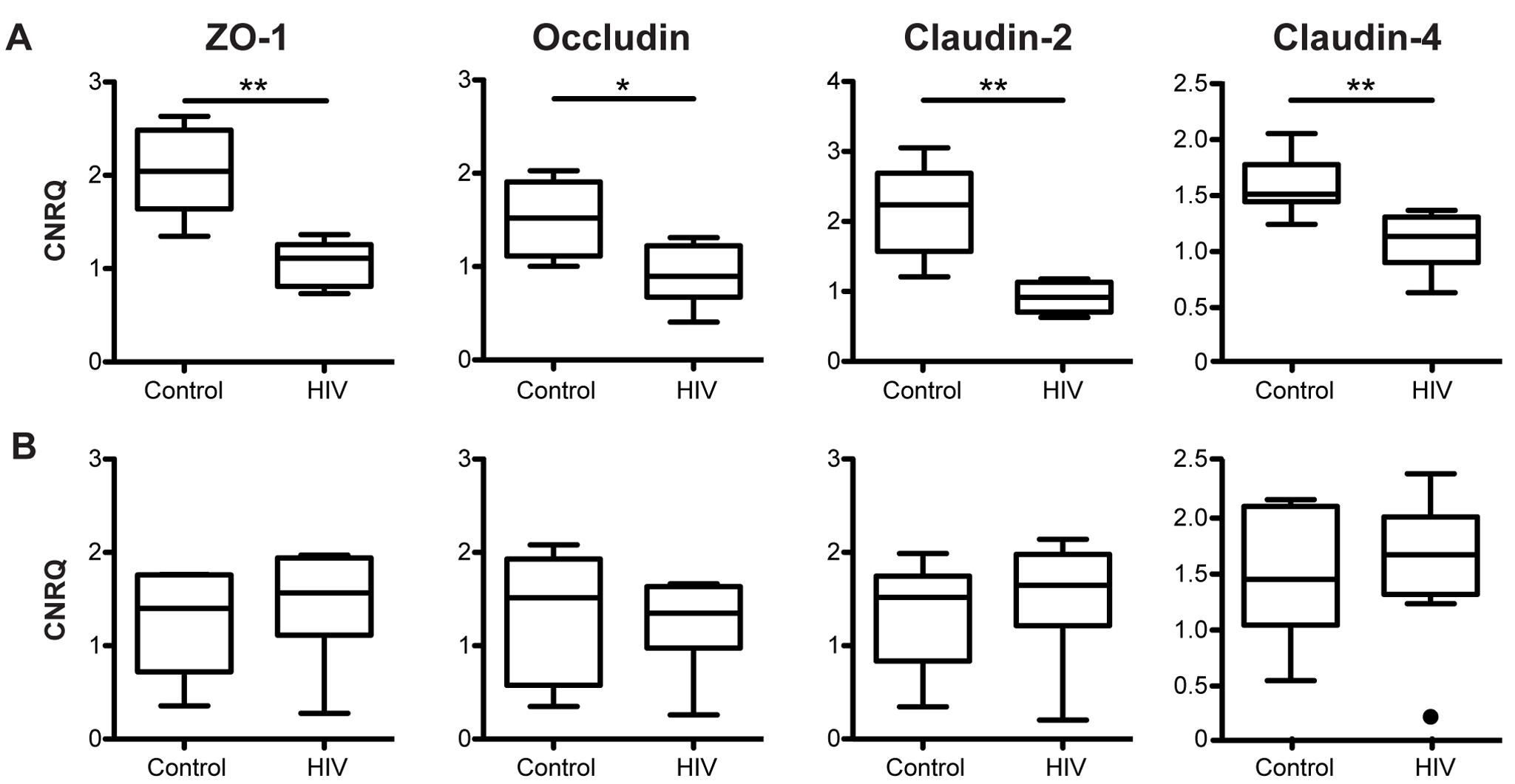

Supplement: Figure S1 — Tight junctional transcripts are decreased in the colon, not the terminal ileum, of male HIV+ individuals. Calibrated normalized relative quantities (CNRQ) of ZO-1, occludin, claudin-2, and claudin-4 transcripts were determined in total RNA isolated from (A) colonic (n = 8 for HIV+, n = 7 for healthy controls) and (B) terminal ileal (n = 8 for HIV+, n = 6 for healthy controls) biopsies of male HIV+ individuals and healthy controls. Levels are normalized to β-actin and eef1α1 expression. Box-and-whisker plots were constructed using Tukey's method, where black dots identify the outliers. Statistical analysis was performed on all data points, including the outliers (* p<0.05, ** p<0.01 between HIV+ and healthy controls). (TIF) [file ppat.1004198.s001.tif]

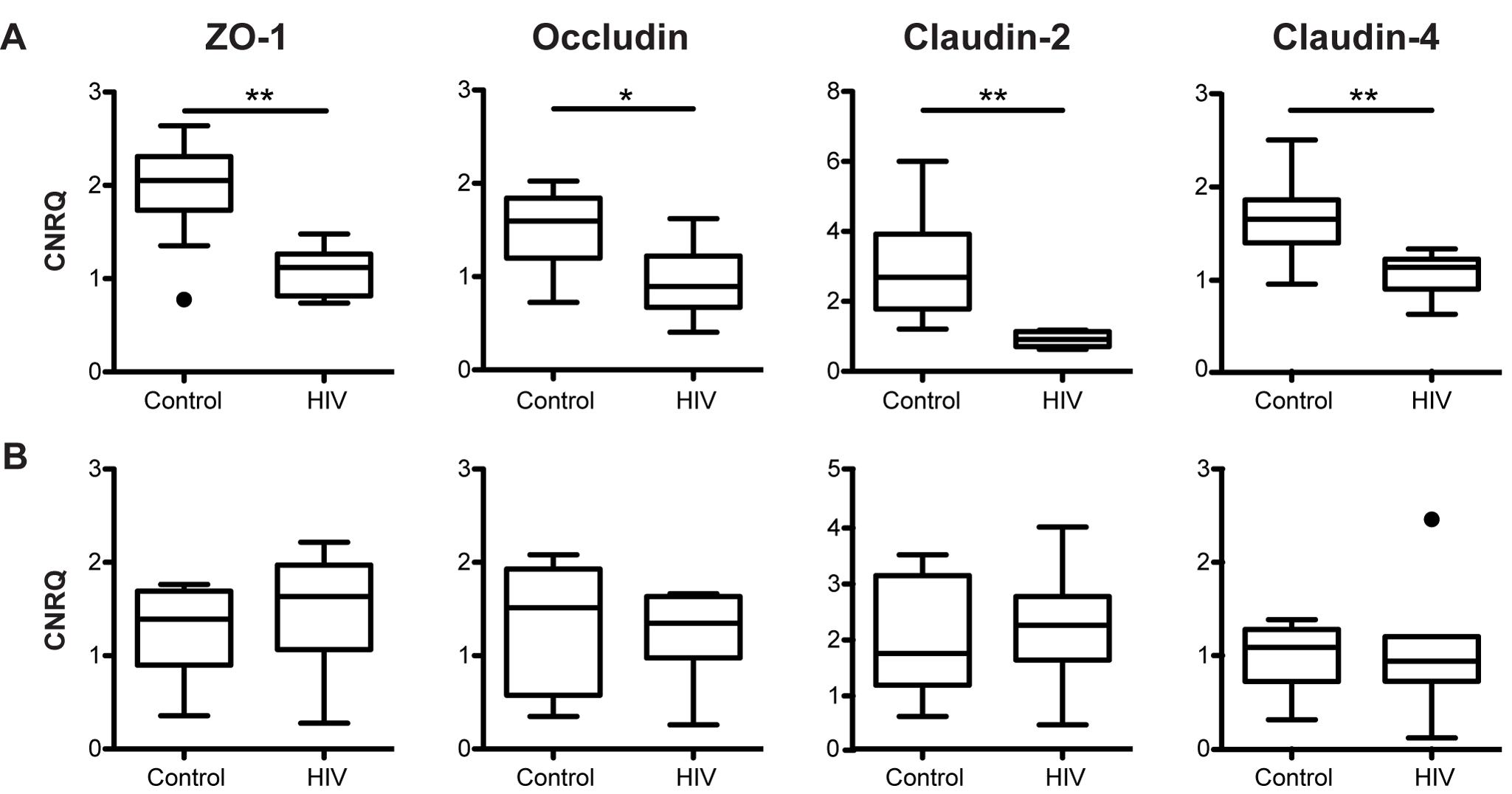

Supplement: Figure S2 — Tight junctional transcripts are decreased in the colon, not the terminal ileum, of virally-suppressed HIV+ individuals. Calibrated normalized relative quantities (CNRQ) of ZO-1, occludin, claudin-2, and claudin-4 transcripts were determined in total RNA isolated from (A) colonic (n = 8 for HIV+, n = 13 for healthy controls) and (B) terminal ileal (n = 7 for HIV+, n = 8 for healthy controls) biopsies of virally-suppressed HIV+ individuals and healthy controls. Levels are normalized to β-actin and eef1α1 expression. Box-and-whisker plots were constructed using Tukey's method, where black dots identify the outliers. Statistical analysis was performed on all data points, including the outliers (* p<0.05, ** p<0.01 between HIV+ and healthy controls). (TIF) [file ppat.1004198.s002.tif]

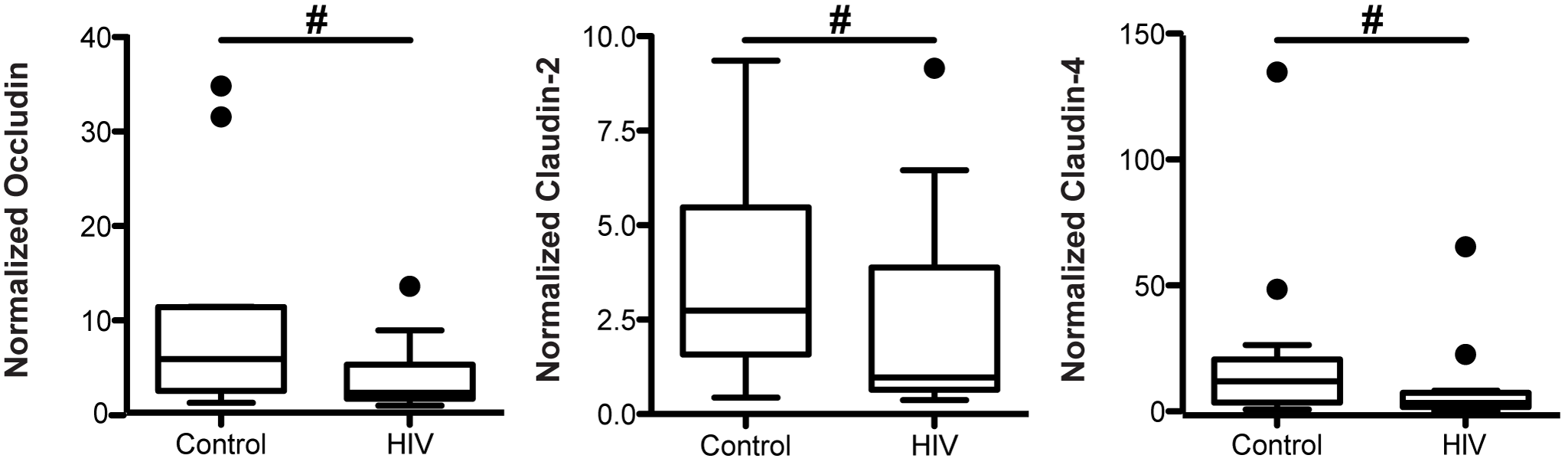

Supplement: Figure S3 — Tight junctional protein levels are decreased in the descending colon of HIV+ individuals. Total protein lysate extracted from descending colonic biopsies of HIV+ individuals and healthy controls were immunoblotted for occludin, claudin-2, claudin-4, cytokeratin-18, GAPDH, and β-actin. Specific bands within the linear density range for occludin, claudin-2, and claudin-4 were quantitated by densitometric analysis, and compared between HIV+ individuals (including those with detectable viral load) and healthy controls. Target protein levels were normalized against cytokeratin-18 protein levels. Box-and-whisker plots were constructed using Tukey's method, where black dots identify the outliers. Statistical analysis was performed on all data points, including the outliers (n = 13 for both cohorts, except n = 12 for healthy controls in claudin-2; # p≤0.07). (TIF) [file ppat.1004198.s003.tif]

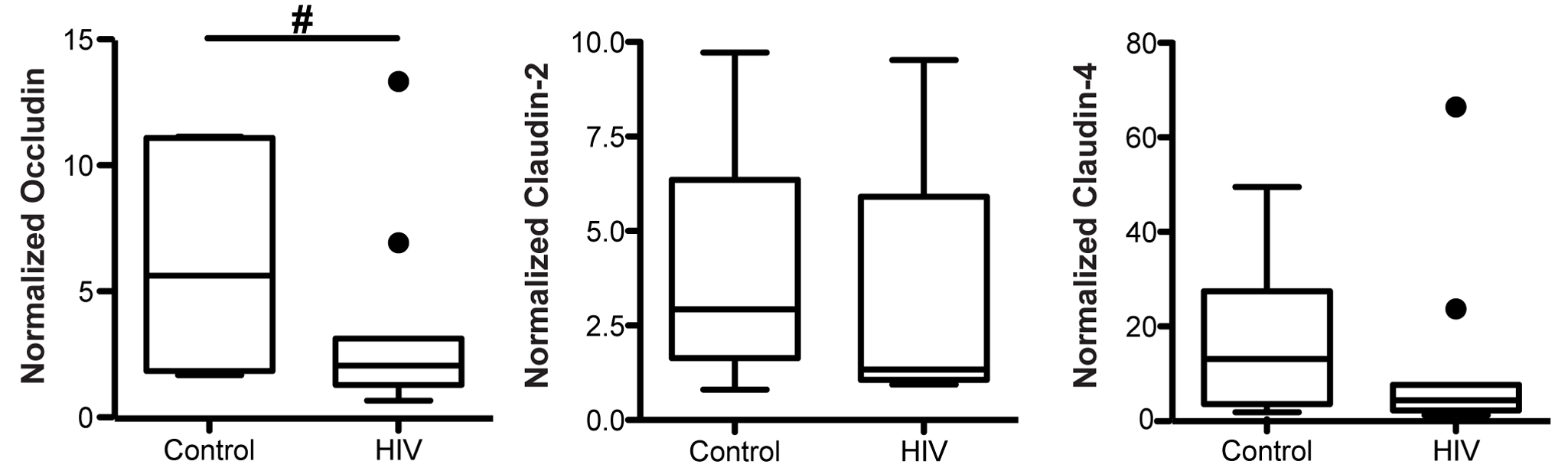

Supplement: Figure S4 — Tight junctional protein levels are decreased in the descending colon of male HIV+ individuals. Total protein lysate extracted from descending colonic biopsies of male HIV+ individuals and healthy controls were immunoblotted for occludin, claudin-2, claudin-4, cytokeratin-18, GAPDH, and β-actin. Specific bands within the linear density range for occludin, claudin-2, and claudin-4 were quantitated by densitometric analysis, and compared between male HIV+ individuals and healthy controls. Target protein levels were normalized against cytokeratin-18 protein levels. Box-and-whisker plots were constructed using Tukey's method, where black dots identify the outliers. Statistical analysis was performed on all data points, including the outliers (n = 11 for HIV+, n = 7 for healthy controls; # p<0.09). (TIF) [file ppat.1004198.s004.tif]

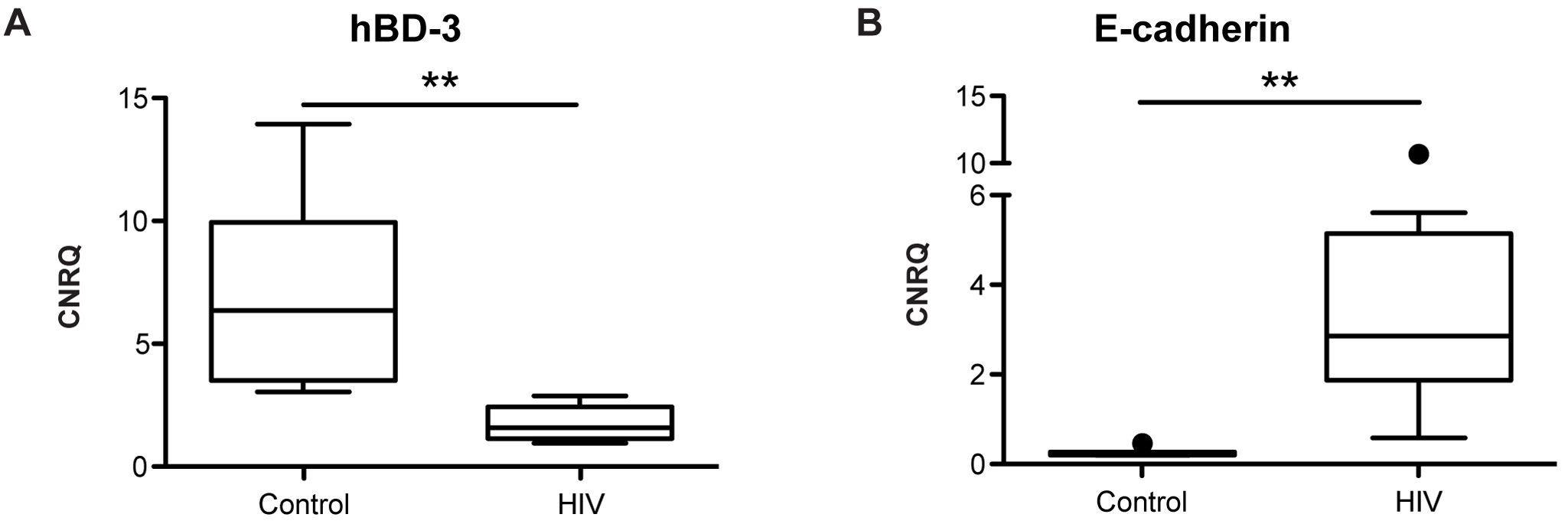

Supplement: Figure S5 — Human β defensin-3 and E-cadherin expression varies differentially in the colon of HIV+ males. Total RNA was isolated from intestinal biopsies, and CNRQ of transcripts for (A) human β defensin-3 (n = 9 for HIV+, n = 7 for healthy controls) and (B) E-cadherin (n = 8 for HIV+, n = 7 for healthy controls) were measured in the colon of male HIV+ individuals and healthy controls. Box-and-whisker plots were constructed using Tukey's method, where black dots identify the outliers. Statistical analysis was performed on all data points, including the outliers. (** p<0.01 between HIV+ and healthy controls). (TIF) [file ppat.1004198.s005.tif]

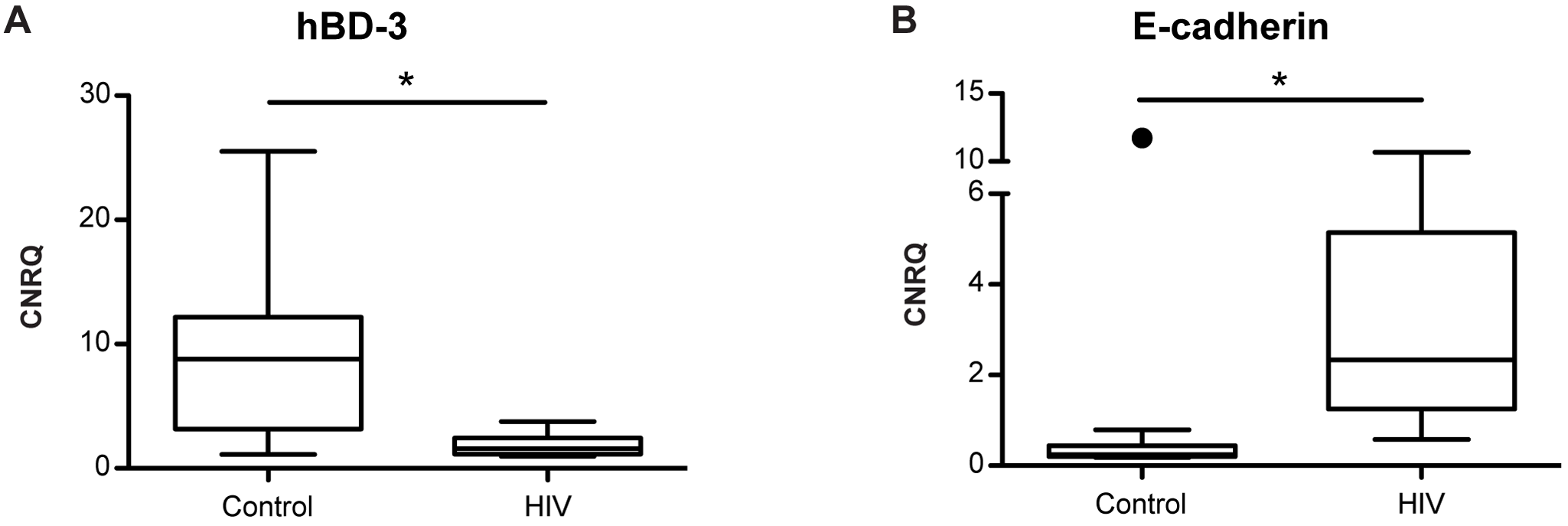

Supplement: Figure S6 — Human β defensin-3 and E-cadherin expression varies differentially in the colon of virally-suppressed HIV+ individuals. Total RNA was isolated from intestinal biopsies, and CNRQ of transcripts for (A) human β defensin-3 (n = 9 for HIV+, n = 12 for healthy controls) and (B) E-cadherin (n = 8 for HIV+, n = 13 for healthy controls) were measured in the colon of virally-suppressed HIV+ individuals and healthy controls. Box-and-whisker plots were constructed using Tukey's method, where black dots identify the outliers. Statistical analysis was performed on all data points, including the outliers (* p<0.05 between HIV+ and healthy controls). (TIF) [file ppat.1004198.s006.tif]
